# Micro- and Nanoplastics and Pulmonary Health: The Current State of Research

**DOI:** 10.3390/microplastics5010029

**Published:** 2026-02-09

**Authors:** Charles E. Bardawil, Jarrett Dobbins, Shannon Lankford, Adam C. Soloff, Rajeev Dhupar

**Affiliations:** 1 Department of Cardiothoracic Surgery, Wake Forest University School of Medicine, Winston-Salem, NC 27157, USA; 2 Department of Cardiothoracic Surgery, University of Pittsburgh School of Medicine, Pittsburgh, PA 15232, USA; 3 Surgical and Research Services Division, VA Pittsburgh Healthcare System, Pittsburgh, PA 15212, USA

**Keywords:** microplastics, nanoplastics, pulmonary health, detection, production

## Abstract

Micro- and nanoplastics are human made environmental contaminants that pose a growing concern for our health, particularly through airborne exposures. Although human autopsy studies confirm that micro- and nanoplastics are retained in lung tissue, our understanding of their short- and long-term effects on the pulmonary system is limited. We reviewed the existing literature to evaluate the effects of micro- and nanoplastics on the respiratory system and how their downstream effects may induce respiratory disease. In vivo and in vitro studies demonstrate that micro- and nanoplastics appear to have the capacity to disrupt pulmonary homeostasis through oxidative stress, immune activation, epithelial remodeling, and surfactant interference. Unfortunately, most available micro- and nanoplastics exposure studies are conducted using environmentally irrelevant plastics at high doses, which limits the accuracy and validity of conclusions regarding biological mechanisms that may contribute to chronic lung disease. To close this gap, future studies must adopt standardized, human-relevant models and realistic exposure scenarios. This includes using advanced in vitro and ex vivo platforms, and environmentally representative micro- and nanoplastics (rather than polystyrene spheres) to improve clinical relevance and support effective prevention and risk mitigation strategies.

## 1. Introduction

The pulmonary system is in constant contact with the outside environment and is susceptible to the effects of airborne pollutants and toxins. It is well established that inhaled toxins lead to lung inflammation, tissue damage, and the development of conditions such as chronic obstructive pulmonary disease (COPD) [1]. The long-term effects can be partially predicted depending on the type of exposure, such as industrial pollutants [2,3] (e.g., diesel fumes), chemicals and toxic gases [3–5] (e.g., chlorine and mustard gas), occupational exposures [6,7] (e.g., asbestos, coal dust, silica), and recreational products such as tobacco smoke and electronic cigarette vapor [8].

In recent years, microplastics (MPs) and nanoplastics (NPs), collectively termed micro- and nanoplastics (MNPs) have become pervasive in our environment and may need to be considered potential toxins. MPs are plastic particles ranging from 1 μm to 5 mm, whereas NPs range from 1 nm to 1 μm [9]. For clarity and consistency, we refer to micro- and nanoplastics (MNPs) when discussing plastic particles in general or when size distinctions are not central to the context. When size-specific data or experimental conditions are relevant, we use MPs or NPs accordingly.

MNPs were raised as a health concern in 2004 by marine biologists at the University of Plymouth in the *Science* article entitled “Lost at Sea: Where Is All the Plastic?”, where the authors addressed the vast extent of MNP contamination [10]. Since that time more attention has been paid to MNP exposures and their potential effects on the environment, marine life, and human health [11–15]. In 2022, the World Health Organization published a report outlining the potential implications of MNPs on human health [15], in which they highlight the respiratory tract as a common route of exposure with an estimated 3000 MNPs being inhaled each day [15,16]. Notably, MNPs of various sizes have been detected in human lungs obtained from autopsies, ranging from 5 to 17 μm. Among the detected polymers, polyethylene (PE) and polypropylene (PP) were the most prevalent [17].

Clinicians, scientists, and the public at large are concerned about the possible impacts of inhaled MNPs. This paper will examine the current landscape of research on the impact of MNPs on the respiratory system. We will briefly go over production methods for research grade MNPs as well as the techniques used for detection in tissue and cell culture. Finally, we will summarize the currently understood pulmonary consequences of MNPs from the existing in vitro and in vivo studies.

## 2. Micro- and Nanoplastics in Research: Environmental Overview and Laboratory Preparation

There is significant heterogeneity of MNP composition in the environment with the primary polymer composition changing dependent on the specific sampling area. A comprehensive review by O’Brien et al. assessed the MNP composition in our atmosphere (from largely residential areas) and found that polyethylene terephthalate (PET), PE, and PP are those which are most commonly found (Table 1) [18].

Polystyrene (PS) spheres are widely used in experimental studies assessing the physiologic and toxicological impact of MNPs, largely because they are commercially available in a range of sizes and fluorescent colors and are commonly used for instrument calibration. However, this widespread use presents limitations. While PS is a known environmental pollutant, it is neither the most abundant nor the most representative MNP found in natural settings, as shown in Table 1, which undermines the ecological and clinical relevance of studies relying solely on them.

Fortunately, other MNPs that are not PS spheres are commercially available (Table 2) or can be produced for more clinically relevant research. A protocol for generating non-PS MP fibers was developed by Cole at the University of Exeter, and it is readily reproducible [19]. This approach has been used in several publications, and our own experience has found this method to be straightforward and reproducible for a number of common MPs [20–23]. Another practical method described by Winkler et al. involves collecting dryer lint after nylon clothes have been cycled, which results in fibers of average size 700 × 10 μm [24]. Both methods enable the production of MP fibers that are relevant to real-world pulmonary exposures.

There are other methods to generate both MP and NP fragments, and most studies describe two strategies: a bottom-up approach involving dissolution followed by precipitation, and a top-down approach that relies on mechanical degradation techniques. For the top-down approach, researchers exploit the fact that plastics become brittle at freezing temperatures, allowing larger pieces to be broken down using a cryomill and then further reduced with ball mills. The resulting fragments are then sieved to remove larger particles that would otherwise be cleared by coughing or the mucociliary escalator [25,26]. Bottom-up approaches involve dissolving polymers in organic solvents under heat, followed by reprecipitation or solidification through anti-solvent addition or emulsion techniques. These methods typically include steps for particle isolation and drying, offering control over size and morphology [27–29].

## 3. Detection of Micro- and Nanoplastics

**Figure 1.**
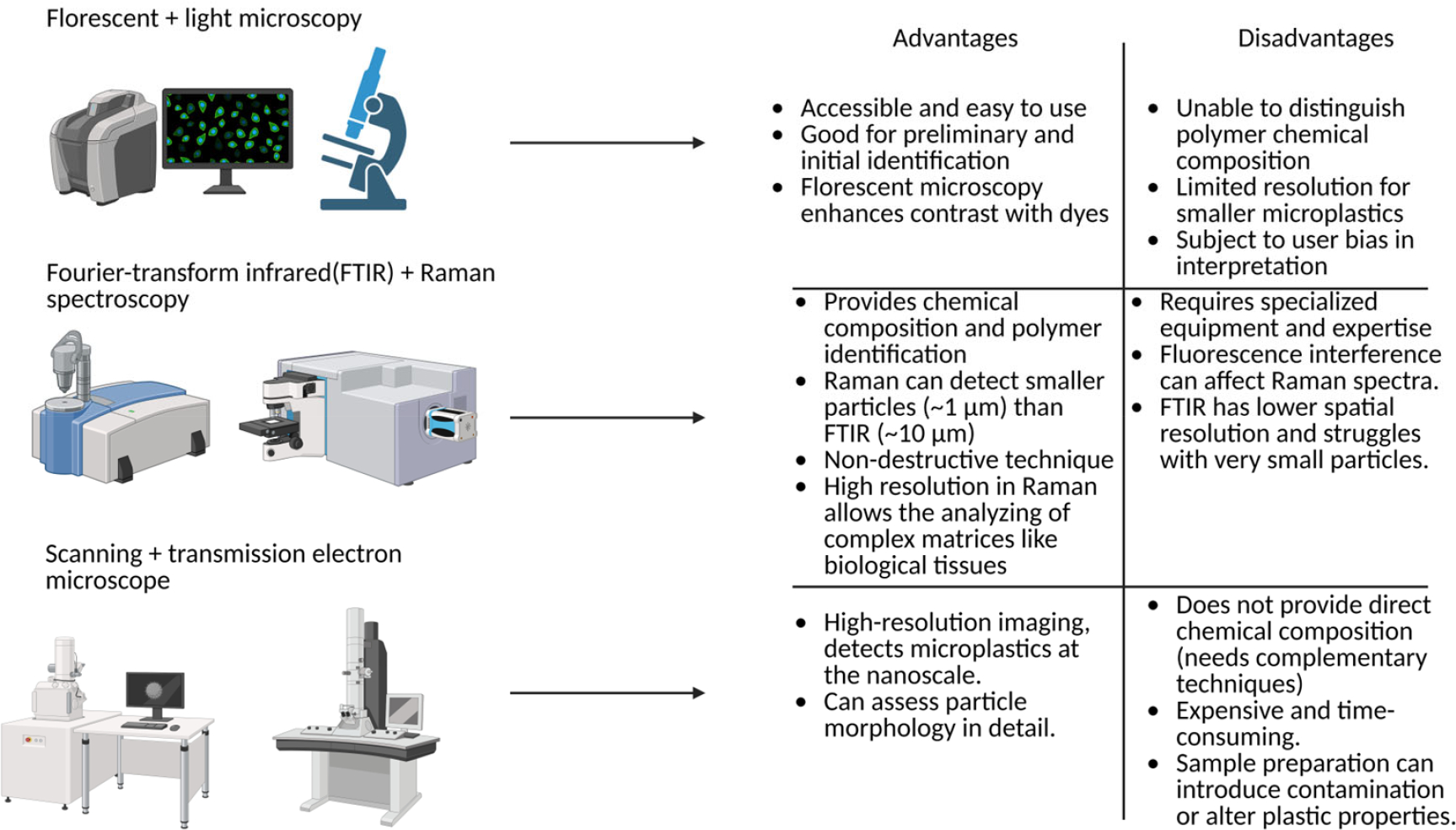
Microscopy, Fourier-transform infrared and Raman spectroscopy, and electron microscopy for detecting micro- and nanoplastics. Comparison of fluorescent/light microscopy, FTIR and Raman spectroscopy, and electron microscopy, highlighting their key advantages and limitations for identifying and characterizing micro- and nanoplastics. Created in BioRender. Charles Bardawil. (2025) https://BioRender.com/phhutxr (accessed on 9 December 2025).

Developing sensitive and quantifiable methods to detect, identify, and characterize MNPs in human lung tissue, particularly in distal regions such as the alveoli, is essential for advancing pulmonary research. Techniques that can distinguish synthetic particles from organic and mineral matter while providing insights into their morphology and chemical composition are important. Among these, microscopy and spectroscopy are the primary methods for visualizing MNPs, with several emerging techniques enhancing detection capabilities (as referenced extensively in other reviews; Figure 1) [30–34]. Without such tools, it remains challenging to assess exposure burden or explore potential correlations between MNP accumulation and respiratory diseases like COPD. Of the available detection tools pertaining to the respiratory system, Fourier-transform infrared (FTIR) and Raman spectroscopy are the most used.

### 3.1. Raman and FTIR Spectroscopy

Spectroscopic techniques, like FTIR and Raman spectroscopy, have become important tools for identifying and characterizing MPs, providing molecular-level insights into polymer composition. FTIR is widely used due to its ability to distinguish MPs from other particulate matter based on the absorption of infrared radiation by molecular bonds which produces a spectrum unique to each polymer type [35]. It is non-destructive and uses attenuated total reflectance to allow the detection of MPs as small as 20 μm without extensive preparation [36,37]. This technique has been used to identify MPs in human lung samples, highlighting potential MP exposure and retention in tissues [38].

Raman spectroscopy offers higher spatial resolution, enabling the analysis of smaller MPs (<10 μm) that are challenging to identify with FTIR [39]. This technique is particularly beneficial for analyzing the complex matrices of biological tissues, and can detect specific polymer types with minimal interference from water and organic materials [40]. The combination of Raman spectroscopy with machine learning and spectral libraries has further enhanced its capabilities, improving throughput and accuracy in polymer identification [41]. Despite these advantages, Raman spectroscopy can be hindered by fluorescence interference and photothermal degradation of polymer particles under high laser power [42].

### 3.2. Microscopic Techniques for Micro- and Nanoplastics Visualization

Optical and stereoscopic microscopy offer accessible, high-resolution imaging for MP morphology but lack the resolution and specificity to identify NPs, especially those relevant to deep lung exposure [30,31,43]. Electron microscopy does provide detailed structural and chemical information compared to light microscopes and can accurately image MPs and NPs in the nanoscale [32–34]. Despite its exceptional resolution, electron microscopy is limited by high operational costs, labor-intensive sample preparation, and a limited field of view that precludes efficient quantification across heterogeneous samples. This limits its practicality for clinical applications. Fluorescence microscopy, which can be used with common dyes such as Nile Red or rhodamine, can localize pre-dyed MPs and NPs in biological tissues and aid in distribution studies for in vitro and in vivo studies [19,44,45]. Unfortunately, MNPs are not naturally fluorescent, which limits the utility of fluorescence microscopy for real-world clinical applications, even though it remains useful for in vitro and in vivo studies.

## 4. Deposition of Mico- and Nanoplastics in the Respiratory Tract

### 4.1. In Silico Modeling of Micro- and Nanoplastic Deposition and Key Influencing Factors

Understanding the patterns of MNP deposition in the respiratory tract is critical to evaluating their potential health impact. Two recent in silico modeling studies have demonstrated that inhaled MPs and NPs exhibit size-, shape-, and breathing rate-dependent deposition behaviors [46,47]. Regarding size, both nanoparticles and nanofibers are more likely to reach the distal airways, while larger microparticles and microfibers are more likely to remain in the nasal passages and oropharynx [46,47].

Additionally, the shape of the particle plays a critical role in respiratory deposition, with cylindrical MNPs exhibiting unique aerodynamic behavior compared to spherical particles. Cylindrical and fibrous MNPs are more likely to bypass upper airway filtration mechanisms and deposit deeper within the bronchial and alveolar regions. This occurs because they undergo more pronounced rotational and tumbling motions, which increase the likelihood of avoiding early deposition in the nasal and oropharyngeal cavities and successfully navigating airway bifurcations. Moreover, a cylindrical shape increases escape fractions, indicating that these particles not only deposit deeper but may also reach distal lung regions and eventually cross the alveolar membranes into the systemic circulation [46,47]. These behaviors are particularly concerning given that cylindrically shaped MNPs are commonly found in household air (e.g., lint from clothing or blankets). Very rarely would a spherical shape be relevant to daily ambient exposures.

### 4.2. Evidence from Human Lung Studies and Knowledge Gaps

While there are only a few studies, research on human lung tissue has revealed limited information about MNP deposits in the respiratory tract. A study using FTIR spectroscopy on peripheral lung parenchyma identified predominantly fibrous MPs ranging from 12 to 2475 μm with a mean width of 22 ± 20 μm [38]. With greater sensitivity, Raman spectroscopy has detected particles smaller than 6 μm and fibers ranging from 8 to 17 μm in proximal and distal lung parenchyma [17]. In sputum samples, FTIR identified MPs with a median particle size of 75 μm (IQR: 45–211 μm) [48]. Studies analyzing bronchoalveolar lavage fluid (BALF) further support pulmonary MNP presence; for example, one FTIR-based report identified MPs of 33 μm in diameter, and another Laser direct infrared imaging (LDIR)-based study showed that approximately 80% of MNPs detected in the lower airways were 20–30 μm in size [49–52]. These studies have demonstrated that there are MNPs in the respiratory system, and that there is a lack of well characterized studies that inform us of how size and shape impact ultimate penetration into the lungs. Given that most detected particles are tens of microns in size, contrary to conventional assumptions that only submicron or nanoscale NPs can penetrate deep into the lungs, there is an urgent need for standardized, high-sensitivity methods capable of detecting particles at or below 1 μm to accurately assess true MNP burden.

## 5. Micro- and Nanoplastic Exposure Models

To investigate the respiratory effects of MNPs, researchers rely on a range of in vitro and in vivo exposure models, each offering unique insights into cellular responses, tissue-level interactions, and systemic outcomes. In vitro studies commonly utilize two main categories of cell models. Primary cells, which include nasal epithelial cells, bronchial and alveolar epithelial cells, and primary macrophages, offer high physiological relevance but limited scalability. Immortalized cell lines, such as BEAS-2B (human bronchial epithelial cells), A549 cells (a human alveolar basal epithelial carcinoma line resembling type II pneumocytes), and RAW 264.7 cells (murine macrophage-like cell line derived from Abelson murine leukemia virus-induced tumors in mice) provide reproducibility and ease of use but are less relevant to actual exposure.

While in vitro models have been instrumental in advancing our understanding of MNP toxicity in the respiratory system, most studies to date have relied on submerged cell cultures. In these models, cells are fully immersed in liquid media containing MNPs, which does not accurately replicate the physiological conditions of the human airway, in which epithelial cells are exposed to air on their apical surface [53]. To address this limitation, various researchers have adopted the air–liquid interface (ALI) model, in which epithelial cells are cultured with their basal side in contact with media and their apical side exposed to air [54,55]. This setup more closely mimics the architecture and function of the respiratory epithelium, allowing for more realistic particle deposition and mucociliary interactions [54,55]. Beyond ALI systems, advanced models such as lung organoids, 3D structures derived from stem cells, offer the ability to study MNP effects in a multicellular, self-organizing context that includes epithelial, mesenchymal, and sometimes immune components [56,57]. Similarly, lung-on-a-chip platforms integrate microfluidics and mechanical forces to simulate breathing motions and vascular flow, enabling dynamic studies toxin translocation, barrier integrity, and inflammatory signaling under near-physiological conditions [58,59]. Together, these emerging models provide more accurate and translationally relevant insights into how MNPs interact with the human respiratory system.

In vivo models play a critical role in evaluating the physiological relevance and systemic impact of MNP exposure on the respiratory system. These models allow researchers to study complex biological responses, including immune activation, tissue remodeling, and particle clearance mechanisms, within the context of intact airway architecture and dynamic breathing. The majority of in vivo experiments are conducted using rodent models with three different exposure methods. The three methods are intranasal, intratracheal, and inhalation-based delivery, each with distinct advantages and limitations. Inhalation exposure most closely replicates real-world human scenarios, especially for studying chronic environmental exposure; however, it is technically demanding, requires specialized equipment, and may pose challenges in dose standardization and reproducibility. Additionally, as outlined in the review by Stucki et al. [60], significant anatomical and physiological differences exist between rodent and human respiratory systems which complicate the extrapolation of inhalation exposure findings, thereby limiting the clinical relevance of such studies. Intranasal exposure similarly mimics natural inhalation, making it suitable for studying environmental exposure and chronic effects, though it introduces variability due to differences in breathing and clearance patterns. In addition, the effects of oropharyngeal absorption, GI tract ingestion, and portal circulation versus systemic circulation entry are not easily accounted for in these models if the desire is to study effects on the respiratory system. Direct intratracheal instillation, in contrast, ensures precise delivery to the lungs, allowing for controlled dosing and reproducibility, which is particularly useful for studying test material that would be difficult to generate at sufficient concentration when aerosolized [61]. Given the anatomical predisposition for MNPs to accumulate in the nasal passages and upper trachea during inhalation or intranasal exposure, direct intratracheal instillation emerges as a more suitable method for investigating toxicity in deeper lung regions. While it sacrifices physiological realism, its precision and reproducibility make it particularly advantageous for studying localized pulmonary effects of MNPs, especially in alveolar and bronchiolar compartments where chronic toxicity may manifest.

## 6. In Vitro and In Vivo Pathological Effects of Micro and Nanoplastics on the Respiratory System

### 6.1. Oxidative Stress and Inflammatory Signaling

#### 6.1.1. Reactive Oxygen Species Generation

A consistent finding across both in vitro and in vivo studies is the induction of reactive oxygen species (ROS) following MP and NP exposure. This effect was observed across different MP and NP subtypes and sizes and occurred in both primary and immortalized cell lines. ROS generation showed a dose-dependent pattern, though even relatively low concentrations were sufficient to trigger oxidative stress [62–75]. ROS generation was induced by internalization of MPs and NPs through integrin α5β1-mediated endocytosis [70] and induced to a greater extent under mimicked breathing conditions [76]. In vivo, intratracheal administration of PP-MPs at doses of 2.5 and 5 mg/kg, and PS-MPs at doses of 0.2 and 1.0 mg/kg, also resulted in ROS production [77,78]. However, ROS production in in vitro studies was not universal. Studies using ALI models, which better replicate physiological conditions, have reported no ROS induction at realistic exposure levels [79]. These findings suggest that submerged culture systems may overestimate oxidative stress responses, highlighting the importance of model selection when evaluating MP and NP toxicity.

The biological consequences of ROS induction extend beyond transient oxidative stress, as these species initiate lipid peroxidation, protein oxidation, and DNA damage, thereby compromising membrane integrity and cellular homeostasis [80,81]. In pulmonary tissues, sustained ROS production following MPs and NPs exposure may activate redox-sensitive transcription factors, leading to pro-inflammatory cytokine release, epithelial barrier dysfunction, and recruitment of neutrophils and monocytes [80]. Over time, this cascade can promote chronic inflammation, fibrotic remodeling, and endothelial injury, underscoring the potential for MPs and NPs to contribute to long-term pulmonary pathology even at sub-toxic exposure levels.

#### 6.1.2. Cytokine and Chemokine Release

Cytokine production has been consistently observed across various in vitro and in vivo models following MP and NP exposure. In cell lines such as BEAS-2B and A549, both MPs and NPs induced expression of key pro-inflammatory cytokines, including IL-1β, IL-6, IL-8, TNF-α, and IL-18, while levels of IL-2, IL-15, and IL-21 were found to be decreased [62,73,77,82–87]. Similar findings were observed in in vivo models, where intratracheal administration of PP-NPs at doses of 2.5 and 5 mg/kg and PS-NPs at 5 mg/kg significantly elevated levels of IL-1β, IL-6, and TNF-α [77,88]. This was accompanied by the persistence of cytokine-induced neutrophil chemoattractants (CINC-1 and CINC-2) for up to one month post-exposure [78,89], as well as sustained elevation of chemokine (C-X-C motif) ligand (CXCL) 1 and CXCL6 mRNA in lung homogenates six months after exposure, suggesting prolonged immune cell recruitment and unresolved inflammation [89]. There were conflicting findings regarding changes in transforming growth factor beta (TGF-β) following NP exposure. One study reported an increase in TGF-β levels [83], while another observed a decrease [85]. Interestingly, TGF-β was found to be reduced in an ALI model using primary human airway epithelial cells, whereas the study showing increased levels utilized submerged A549 cells, stressing the influence of model type on immunoregulatory outcomes. Two separate studies investigated the effects of various MPs and NPs at realistic exposure doses using different culture models. In an ALI model employing A549 and BEAS-2B cells, no changes in cytokine levels were observed across MP and NP types [90]. In contrast, a submerged culture study using the same cell lines reported an increase in IL-8 specifically in the polyamide group [91].

#### 6.1.3. Toll Like Receptor Activation

Toll-like receptors (TLRs) are key pattern recognition receptors that detect pathogen- and damage-associated molecular patterns, initiating innate immune responses. Upon activation, TLRs trigger downstream signaling cascades, including the NF-κB pathway, which lead to the transcription of proinflammatory cytokines, chemokines, and inflammasome components, thereby shaping both acute and chronic inflammatory responses [92,93]. Intratracheal exposure to PS-MPs has been shown to upregulate TLR2 and TLR4 protein levels in whole lung homogenates, as demonstrated by Western blot analysis [74,87,94]. However, findings vary across studies: one reported that PS-MPs increased TLR4 and NLRP3, but not TLR2, while PP-MP exposure selectively upregulated TLR2 without affecting TLR4 [95]. These findings were concurrent with elevated levels of NF-κB as well. These changes may contribute to downstream immune activation, including macrophage recruitment and polarization, which are central to both acute defense and chronic lung remodeling.

The observed upregulation of TLR2 and TLR4 following MP and NP exposure may reflect a conserved innate immune response similar to that triggered by other airborne toxicants. TLR4 is known to recognize diesel exhaust particles, mediate cytokine production in response to cigarette smoke, and contribute to oxidant-induced lung injury, while TLR2 is upregulated in COPD patients and responds to hyaluronan fragments during lung injury [93,96,97]. These parallels suggest that MPs and NPs may engage TLRs in a manner akin to established environmental pollutants.

### 6.2. Immune System Activation

#### 6.2.1. Macrophages

Alveolar macrophages play an essential role in defending against inhaled pathogens and toxins by virtue of being one of the first immune cells to encounter them. As the “sentinels of the lung”, they readily phagocytose pathogens and recruit immune cells through both antigen presentation, cytokine production, and chemokine release [98]. Data is mixed on the effect of MPs and NPs on macrophages, due to the vast array of MP and NP composition, size, and shape, along with differences from using cell lines versus primary macrophages. In terms of size, when comparing PS-MPs from 1 to 6 μm, macrophages seem to phagocytose those between 2 and 3 μm the quickest [99]. Additionally, shape may play an important role as well. For example, rod-shaped NPs at 0.5 μm are more readily taken up by macrophages compared to spheres, whereas at 3 μm spheres were more readily taken up than rods [100]. However, macrophages have limited capacity to engulf larger particles, leading to frustrated phagocytosis of needle-shaped microfibers greater than 15 μm and microspheres exceeding 10 μm [101]. Primary macrophages are capable of engulfing PS spheres, possibly facilitated by T-cell membrane protein 4 (Tim4)-mediated binding [102], and can phagocytose PS spheres up to 10 μm in size; however, the spheres were unable to be degraded after 72 h [103]. PS-MP and NP spheres have been shown to influence macrophage metabolic activity in a dose-dependent manner, shifting energy production from mitochondrial respiration to increased glycolysis. While the PS-MPs and NPs did not induce ROS production, they led to elevated nitrite and lactate dehydrogenase levels, with the PS-NPs (0.5 μm in size) exerting the most significant effect [104]. Additionally, polyamide fibers have been found to upregulate TLR4 gene expression [22]. This was in contrast to 1.1 μm polymethyl methacrylate fibers (100 μg/mL) decreasing pro-inflammatory activation markers in primary macrophages and promoting an M2 phenotype [105]. MP and NP exposure additionally led to marked cytotoxic and pro-inflammatory effects, including reduced cell viability, increased ROS generation, and mitochondrial dysfunction [78,106]. These changes were accompanied by upregulation of Matrix Metalloproteinase (MMP)-9, MMP-12 and inflammatory mediators (IL-1β, TNF-α, IL-6, NLRP3), suggesting enhanced tissue remodeling and inflammasome activation [107,108].

In vivo studies further support the involvement of alveolar macrophages in MP and NP uptake and retention. Intratracheal administration of PP-MPs at 4 mg/kg for three weeks, as well as aerosolized exposure at 10 mg/m^3^, resulted in internalization of MPs within alveolar macrophages, detectable in fixed lung sections even three months post-exposure [89]. Similarly, intratracheal exposure to 0.67 μm PP-NPs at 5 mg/kg for four weeks led to inflammatory cell infiltration and the formation of “foamy macrophage” aggregates in lung tissue [77]. Comparative studies using PE, PS, and PET at 2 mg/kg intranasally confirmed their capacity to trigger immune cell infiltration [109]. Additionally, tire-derived NPs (100 nm at 1 mg/kg) were associated with increased macrophage accumulation in fixed lung sections, as visualized by microscopy [110]. The findings of “foamy macrophages” and internalized MPs and NPs, even 3 months post exposure raise concerns about the long-term consequences of MP and NP accumulation in pulmonary immune cells. Foamy macrophages, which are lipid-overloaded macrophages, may play a central role in the pathogenesis of chronic lung disease by sustaining fibrotic signaling through M2 polarization and persistent TGF-β1 production in response to oxidized phospholipid accumulation [111]. Indeed, macrophages undergo metabolic reprogramming shifting their phenotype dependent on the local niche [112]. Finally, pulmonary toxin exposure typically triggers an influx of pro-inflammatory M1 macrophages at injury sites, followed by recruitment of anti-inflammatory M2 macrophages involved in tissue repair [113,114]. The pathological outcome depends on the balance between these macrophage subtypes, as excessive activation of one or the other can lead to acute lung injury or chronic lung diseases due to overproduction of mediators like ROS, MMPs, and growth factors [113–115]. Perhaps MPs and NPs alter the balance between pro-inflammatory and pro-fibrotic signaling cascades, with lipid-rich microenvironments driving a sustained M2-like state that exacerbates tissue remodeling and chronic inflammation.

#### 6.2.2. Neutrophils

Neutrophils play a vital role in the lungs. They are rapidly recruited from the systemic circulation in response to inflammatory stimuli, including infection, injury, and inhaled toxicants. Upon arrival, neutrophils contribute to host defense through the release of ROS, proteases, and neutrophil extracellular traps, which help neutralize pathogens and clear debris [116]. However, their potent effector functions can also disrupt epithelial integrity, increase alveolar-capillary permeability, and promote inflammation and tissue damage, and eventually chronic lung disease, if not tightly regulated [117,118]. As described above MPs and NPs induce increased recruitment of neutrophils through elevated levels of neutrophil chemoattractants such as CINC-1, CINC-2 (rodent analogs of chemokine (C-X-C motif) ligand 1 and 2) and chemokine ligand 3, implicating neutrophils as key mediators of acute inflammation [78,85,89]. Additionally, studies investigating BALF found increased white blood cell and neutrophil count post MP and NP exposure [86,95,119,120].

### 6.3. Cell Fate and Damage

#### 6.3.1. Mitochondrial Dysfunction

MPs and NPs of different sizes and composition were found to affect mitochondrial function in both in vitro and in vivo experiments. In human nasal epithelial cell cultures, exposure to NPs of 50 or 500 nm reduced mitochondrial membrane potential [72,75]. MPs and NPs were also found to impact the mitochondria of BEAS-2B, A549 and human pulmonary alveolar epithelial cells with a reduction in mitochondrial function and energy generation [64,66,67,71,84,121,122]. More specifically, seahorse assays identified decreases in basal and maximal respiration, ATP production, and spare respiratory capacity in airway epithelial cells [67,123]. Interestingly, in primary macrophages, exposure to PS MPs greater than 1 μm resulted in decreased oxidative phosphorylation but increased glycolysis and overall metabolic activity, suggesting substantial energy expenditure and the induction of an immunometabolic state aimed at combating MPs [103,104].

#### 6.3.2. Cellular Senescence

Exposure to MPs and NPs have been shown to induce cellular stress in mononuclear phagocytes, promoting a senescent phenotype characterized by impaired proliferation, evidenced by significantly elevated Senescence-Associated β-Galactosidase and p21 levels [62–64,86,122,124–127]. This is important as recent research has identified early-onset cellular senescence as a hallmark of chronic lung diseases, particularly COPD [128–130], raising the possibility that MP and NP-induced senescence may contribute to disease pathogenesis. Senescent cells, while no longer dividing, remain metabolically active and initiate a pro-inflammatory secretory phenotype. This includes the release of cytokines and chemokines (senescence associated secretory proteins) that activate NF-κB signaling, which in turn amplifies the production of additional inflammatory mediators such as MMPs, ROS, and growth factors. This self-reinforcing inflammatory loop may play a critical role in driving chronic tissue damage and remodeling observed in COPD [131].

#### 6.3.3. Genotoxicity

Genotoxicity was rarely explored as an endpoint in different exposure models. Two in vitro studies have examined the effects of chronic NP exposure and found that NPs induced significant genotoxicity over time, including DNA strand breaks [79,132]. In the first study there was only significant genotoxicity upon 2 weeks exposure [79] whereas in the second study, genotoxic effects were evident only after 30 weeks of exposure, not at the intermediate 15-week time point [132]. Genome instability and more recently cellular senescence through the secretion of senescence associated secretory proteins have been heavily implicated in the mechanisms of cancer development [133], which raises the question of whether long-term, low-dose exposure models may be more appropriate for studying the in vitro effects of MPs and NPs particularly in the context of genotoxicity and potential carcinogenesis.

#### 6.3.4. Cell Viability

The ability of MPs and NPs to induce cell death has been extensively investigated across numerous studies. Although there exists many conflicting results, the general consensus is that MPs and NPs do not cause significant cell death unless administered at extremely high concentrations (up to 1000 μg/mL). Even at these levels, the reduction in cell viability was not a major one [63,65,67,69,82–84,87,125,134]. This trend was observed among various cell types. Most of those studies utilized commercially purchased PS spheres and one important caveat is the availability of sodium azide in many of their formulations. In acute toxicity tests on Daphnia magna (freshwater filter feeders) using commercial PS-NPs (20 nm and 200 nm) containing sodium azide, researchers compared nondialyzed particles, dialyzed particles (sodium azide removed), and sodium azide alone to assess mortality and swimming behavior. The study found that the acute toxicity of the complete PS-NP formulation was primarily due to sodium azide rather than the particles themselves, as dialyzed particles caused no mortality compared to nondialyzed particles [135]. Although this experiment was conducted on an aquatic organism, its findings may be extrapolated to in vitro cell line studies and underscore the challenges of drawing definitive conclusions when using commercially sourced plastics containing preservatives. Finally, as highlighted in this review by Petersen et al., commercially available MP and NP dispersions often contain antimicrobials, surfactants, and other additives, which can introduce extensive artifacts, biases, and misinterpretations in toxicity assessments by confounding observed effects with those of the formulation components rather than the particles themselves [136].

#### 6.3.5. Ferroptosis

Multiple studies have investigated ferroptosis, a type of programmed cell death dependent on iron, as a potential mechanism of MP- and NP-induced pulmonary toxicity in rodent models. Ferroptosis may play a role in the development of chronic lung diseases, with an increasing body of research exploring therapeutic targets for various lung diseases [137,138]. In C57BL/6 mice, intratracheal exposure to 100–200 nm PS-NPs for one week (12.5–25 mg/kg) significantly increased Hypoxia inducible factor-1α and 1β protein levels, along with elevated iron and malondialdehyde, markers indicative of ferroptosis [139]. Similarly, 20 nm PS administered at a dose of 10 mg/kg led to downregulation of ferritin heavy chain 1, resulting in iron overload and potential ferroptosis [140]. Another study using an inhalation chamber exposed mice to four different NPs (PS, PVC, PET, and PE) at a dose of 1 mg/m^3^ for 4 h daily over 14 days. Proteomic analysis identified ferroptosis as a significantly enriched pathway, evidenced by increased Fe^2+^ and MDA levels and reduced glutathione peroxidase activity in whole lung homogenates [120]. A similar inhalation study using 40 nm PS at a daily dose of 100 μg for three months confirmed these findings, reporting decreased ferritin, increased Fe^2+^ and MDA, and development of COPD-like lung damage [141]. There have been attempts to establish a link between COPD and ferroptosis. In humans, a prospective observational study comparing patients with COPD to smokers without the disease found significantly higher serum levels of soluble transferrin receptor 1 and lower levels of glutathione peroxidase in the COPD group [142]. Additionally, the soluble transferrin receptor 1/ glutathione peroxidase ratio was markedly elevated, suggesting a potential imbalance in ferroptosis-related pathways associated with COPD.

### 6.4. Epithelial and Tissue Remodeling

#### 6.4.1. Epithelial Barrier Disruption

In a “lung on a chip” co-culture model that used primary alveolar cells, phorbol myristate acetate differentiated THP-1 cells, and human endothelial cells, 41 nm PS-NPs was found to reduce the barrier resistance and cross the alveolar-capillary barrier and enter the circulation of the system compared to negative control [59]. In Calu-3 ALI model, Polylactic NPs additionally increased its barrier’s permeability indicating barrier damage [79].

#### 6.4.2. Epithelial-to-Mesenchymal Transition and Epithelial Remodeling

Both PE-MPs and PE-NPs altered cellular morphology and induced modulation of epithelial–mesenchymal transition (EMT) markers, including β-catenin and vimentin in BEAS-2B cells. These changes were associated with enhanced migratory capacity rather than invasive behavior [67,143]. This effect was reverted upon removal of the plastics. An in vivo study exposing mice to MPs and NPs for 60 days found E-cadherin levels to be significantly decreased while vimentin was increased [106]. One possible mechanism might be through NADPH oxidase 4 upregulation as seen when NPs were also found to induce epithelial-to-mesenchymal transition in A549 cells [144]. Induction of EMT is particularly significant because it is implicated in the pathogenesis of non-small cell lung cancer and COPD. Notably, reduced E-cadherin expression, a hallmark of EMT, has been correlated with cigarette smoke exposure and is frequently observed in both COPD and lung cancer [145,146].

Winkler et al. developed an airway organoid by isolating tissue-resident stem cells derived from human lung tissue taken during surgery. The tissue was made into a single cell suspension and then suspended in Matrigel and allowed to solidify, enabling the formation of organoid resembling human airway epithelium. To introduce MPs into the organoids, PET fibers collected from the exhaust air of a domestic tumble dryer were resuspended in culture medium and sonicated for uniform dispersion. The organoid fragments were incubated with PET, and this was then encapsulated in Matrigel droplets. They found that the PET fibers were incorporated into the organoids, with some organoids growing in a polarized fashion around the fibers, forming a layer that enveloped the fibers. This raises the issue on the potential for fibers to be chronically retained in human lungs and become encapsulated [24]. In a separate but similar organoid model, exposure to polyester or nylon fibers was by either directly incubating with the Matrigel or being placed on top of the Matrigel. After 21 days, the nylon fibers were found to inhibit the growth of lung organoids while polyester fibers mainly affected epithelial differentiation. The authors conclude that this may be related to alteration of Hox family signaling [20].

#### 6.4.3. Fibrosis and Extracellular Matrix Deposition

MPs and NPs induced fibrosis and extracellular matrix (ECM) deposition in both in vitro and in vivo studies. A549 cells exposed to MPs for two days and then co-cultured with fibroblasts, resulted in increased collagen formation compared to controls, an effect not observed when fibroblasts were cultured with MPs alone [147]. Additionally, MPs and NPs of various subtypes were also found to upregulate markers of fibrosis such as actin, vimentin, and collagen [87,143,147]. In vivo studies consistently demonstrate that MP and NP exposure alters lung architecture, leading to dose-dependent collagen deposition and fibrosis. Most studies utilized repeated exposure methods to deliver MPs and NPs into the lungs. Repeated exposures, such as 5 μm PS-MPs at 6.25 mg/kg three times per week for three weeks or daily exposure to 5 mg/kg PS for two weeks, increased collagen deposition and α-SMA expression [147]. Longer-term models include 3 μm PS-MPs at 30 or 300 μg intratracheally every two weeks for eight weeks [94], 5 μm PS-MPs at 15 mg/kg intratracheally every five days for 60 days [74], and 100 nm PS-NPs at 2 mg/m^3^ via inhalation chamber for 90 days [107]. These studies reported increased fibrosis in high-dose groups, with elevated collagen, actin, and MMP expression. Mechanistically, fibrosis may involve macrophage-mediated TNF-α secretion activating the NLRP3/MMP-9 pathway, leading to ECM degradation and alveolar structural damage [73], as well as cGAS/STING pathway activation, which promotes inflammation and cell injury [108].

In direct size comparison studies, intranasal administration of PS-MPs showed that smaller particles (1–5 μm) induced greater fibrosis than larger ones (10–20 μm) [87]. Another study exposed male mice using an inhalation chamber to 1.5 × 10^5^ particles/m^3^ of either 80 nm PS-NPs or 1 μm PS-MPs for 60 days. While the 80 nm NPs reached extrapulmonary organs more readily, the 1 μm group exhibited more severe pulmonary pathology, including increased fibrosis as seen by trichome staining, pronounced tracheal wall damage, and pronounced structural deterioration [106]. These findings are likely due to deeper lung deposition of smaller particles, as demonstrated in another study where the 100 nm PS-NP group had the highest particle deposition compared to the 500 nm, 1 μm, and 2.5 μm groups [148]. This effect is amplified by the higher particle number and greater surface area of smaller particles, which enhance tissue interactions and cellular uptake. Other polymers, including PP, PE, and PET, also disrupt lung architecture; for instance, intratracheal administration of 0.7 μm PP-NPs at 5 mg/kg for four weeks caused alveolar epithelial hyperplasia [77], and a comparative study of PE, PS, and PET (2 mg/kg intranasally) confirmed their ability to induce fibrosis [109]. Progressive fibrosis and extracellular matrix remodeling ultimately impair lung compliance and gas exchange, leading to reduced pulmonary function and worsening testing outcomes as discussed below.

#### 6.4.4. Altered Pulmonary Function Testing

Several studies have evaluated the functional consequences of MP and NP exposure on pulmonary performance. In rodent models, high-dose PS-MP and PS-NP exposure significantly reduced lung function as measured by non-invasive whole-body plethysmography. Specifically, increased respiratory rate [74], heightened airway hyperresponsiveness [127], and reduced inspiratory time [74,149] were reported. Similarly, tire-derived NPs reduced forced vital capacity and forced exhaled volume when assessed [110]. In one extended exposure model (3 μm PS-MPs at 30 or 300 μg every two weeks for eight weeks), mice exhibited a statistically significant reduction in all tested pulmonary function tests at the highest dose [94]. Collectively, these findings indicate that structural changes such as fibrosis and ECM remodeling are accompanied by measurable declines in respiratory function, reinforcing concerns about chronic inhalation exposure to MPs and NPs.

### 6.5. Lung Surfactant Dysfunction

Computational molecular modeling performed using PP and PVC showed that NPs can adsorb components of the lung surfactant, forming a biomolecular corona that alters their surface properties and renders them hybrid particles composed of both synthetic polymer and endogenous surfactant [150]. This phenomenon is analogous to the formation of a protein corona, a dynamic layer of biomolecules, primarily proteins, which adsorb onto the surface of nanoparticles upon exposure to biological fluids. The resulting corona alters the particle’s identity, influencing its biological interactions, cellular uptake, and toxicity profile. In the case of airborne MPs and NPs, surfactant adsorption may similarly transform their surface properties, potentially modulating their behavior in the respiratory tract and contributing to pathophysiological effects. This effect was shown in vitro when PS-MPs and PS-NPs increased the surface tension of surfactant by interfering with the surfactant’s lipid bilayer and altering its membrane structure [151,152]. PS-MPs and PS-NPs additionally reduce levels of surfactant protein A and B, which are crucial for lamellar body formation and surface tension regulation [83,153]. Finally, when the PS spheres were “aged” by exposing them to direct sunlight or UV light, there was increased free radical formation, which caused lipid peroxidation and protein damage in a dose dependent effect [126,151].

### 6.6. Impact on Pre-Existing Disease

There are suggestions that MPs and NPs may exacerbate pre-existing respiratory conditions such as asthma or increasing susceptibility to acute infections. In one study, asthmatic mice administered 1–5 μm PE microspheres intranasally at 300 μg daily for four weeks exhibited greater goblet cell hyperplasia, collagen deposition, inflammatory cell infiltration, and Th2 immune polarization compared to asthmatic mice not exposed to MPs. These changes were accompanied by increased oxidative stress, elevated IL-33 levels in BALF and more pronounced disruption of airway epithelial barrier integrity and permeability [154]. Similar findings were observed using nylon fibers, with the additional effect of increased airway sensitivity during methacholine challenge testing [155]. One proposed mechanism involves activation of the PI3K-Akt-mTOR pathway, whereby MPs and NPs stimulate airway epithelial cells to secrete heat shock protein 90 alpha, promoting airway smooth muscle cell proliferation and contributing to airway narrowing, hyperresponsiveness, and exacerbation of asthma symptoms [156]. Finally, intranasal exposure to 5 μm PS-MPs at 2.4 mg resulted in significantly greater macrophage–MP aggregates in asthmatic mice compared to non-asthmatic controls, suggesting that MPs may induce persistent immune alterations in individuals with chronic airway inflammation [157]. PS-NPs also influenced the ability for influenza A to infect A549 cells by inhibiting the activation of Tank Binding Kinase1 phosphorylation and down-regulating the production of Interferon-β [158]. Detailed study characteristics for in vitro and in vivo models are summarized in Tables 3 and 4.

## 7. Discussion and Conclusions

The impact of MNPs on human health is drawing increased attention. We have long known that inhaled substances such as tobacco smoke, burn-pit hazards, asbestos, or coal dust impact our health and influence the development of obstructive and restrictive lung disease or cancer. However, we may be missing some of the most pervasive exposures that are inside and outside our homes.

**Figure 2.**
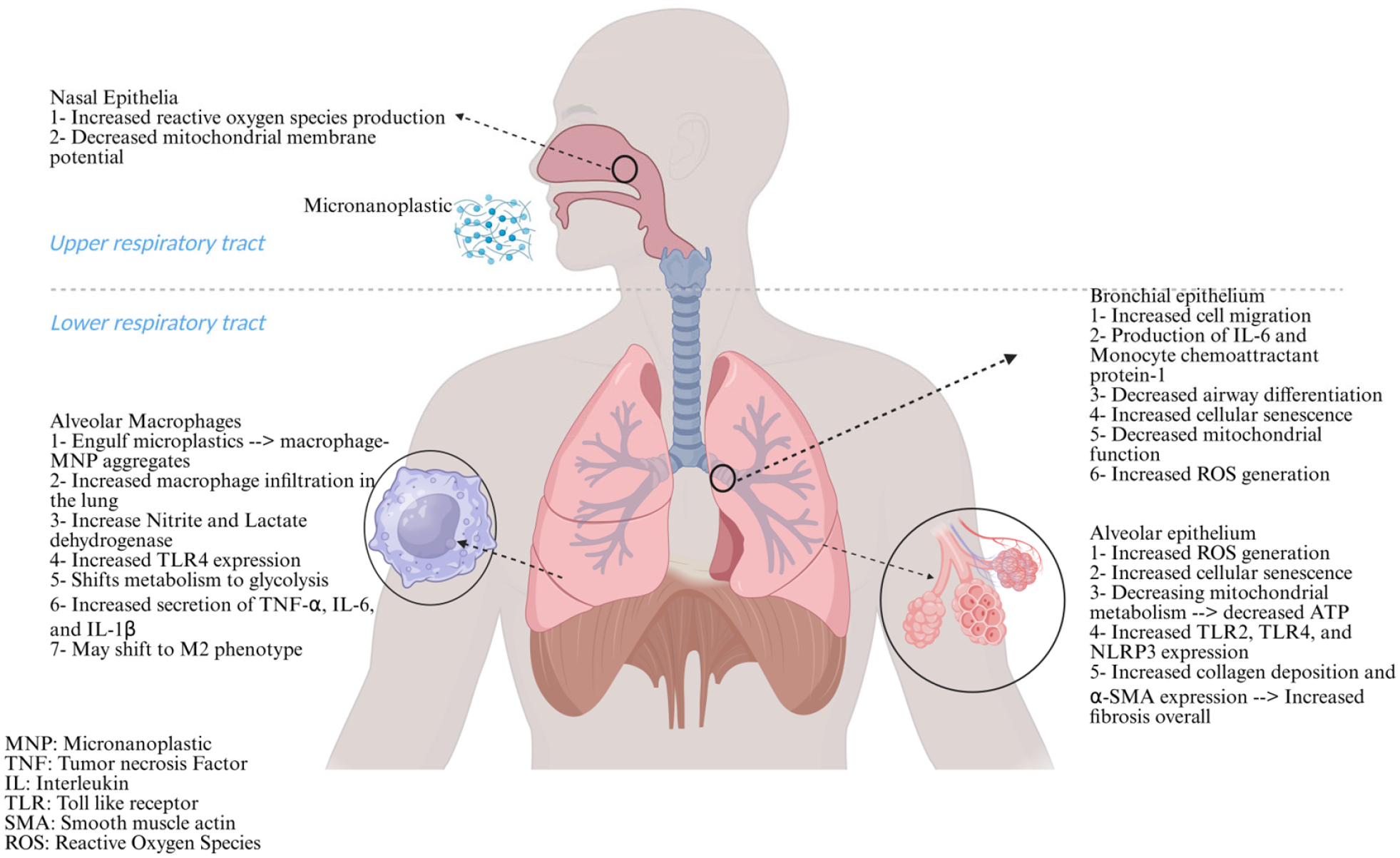
Effects of micro-and nanoplastics on the pulmonary system based on both in vitro and in vivo Studies. Schematic representation of the respiratory system highlighting the cellular and molecular effects of micro- and nanoplastic exposure across nasal, bronchial, and alveolar regions. Created in BioRender. Charles Bardawil. (2025) https://BioRender.com/qg2js4o (accessed on 9 December 2025).

Across many models, our review identifies a pattern of sub-lethal but mechanistically consequential effects of MNPs on the lung (Figure 2). In vitro, submerged epithelial systems repeatedly show oxidative stress, mitochondrial dysfunction, pro-inflammatory signaling, and cell-fate shifts such as senescence, with overt cytotoxicity uncommon except at supraphysiological concentrations. More physiologically relevant platforms, including air–liquid interface, organoids, and lung-on-chip systems, generally trend toward less overt cytotoxicity and inflammation at realistic surface doses, though they still reveal important phenotypes such as barrier disruption, altered epithelial differentiation, and particle translocation across the alveolar–capillary interface. MNPs have been shown to interact directly with pulmonary surfactants, increasing surface tension and altering its biophysical properties, which may compromise alveolar stability and gas exchange. Unfortunately, most in vitro study findings are hampered by dose non-equivalence and formulation confounders in commercial PS-MNPs, reinforcing the need for further experiments using environmentally realistic materials.

Animal studies corroborate these mechanistic signals while highlighting systems-level consequences. Acute and sub-chronic exposures consistently activate TLR, NF-κB, and inflammasome pathways, drive macrophage and neutrophil accumulation, disrupt surfactant biology, and culminate in fibroproliferative remodeling. Select models document functional impairment which include airway hyperresponsiveness, altered respiratory timing, and declines in pulmonary function, especially with repeated or longer exposures. Additionally, particle size influences pulmonary responses with NPs, often causing greater oxidative and inflammatory signaling compared to MPs. At the same time, most in vivo work still relies on high doses and PS-dominant test materials, leaving true chronic, low-dose inhalation underrepresented.

**Figure 3.**
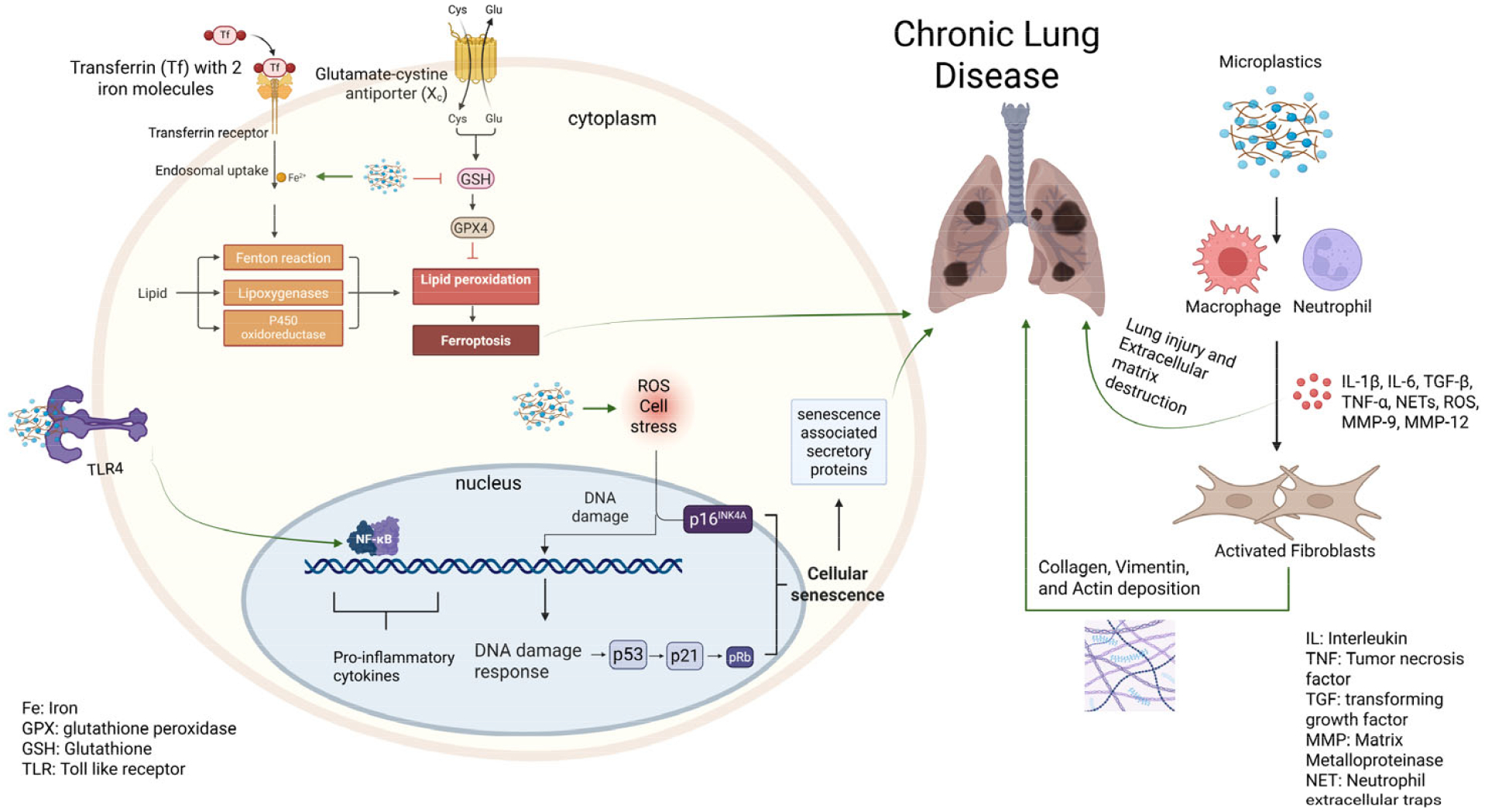
Proposed mechanisms for micro- and nanoplastic-induced lung disease. Micro- and nanoplastics (MNPs) deposit on the airway epithelium and activate immune cells (macrophages, neutrophils), releasing inflammatory mediators (IL-1β, IL-6, TNF-α, TGF-β, ROS, NETs, MMP-9, MMP-12). These signals drive fibroblast activation and extracellular matrix remodeling, resulting in lung injury and fibrosis. Concurrently, MNP exposure disrupts iron homeostasis via transferrin uptake and promotes oxidative stress through lipid peroxidation and ferroptosis. Persistent oxidative stress leads to DNA damage and cellular senescence (p53, p21, CHK1, p16ˆINK4A), leading to the production of senescence associated secretory proteins and ultimately chronic lung disease. Overall, MNPs act as a primary trigger for lung injury, inflammation, and fibrosis. Created in BioRender. Charles Bardawil. (2025) https://BioRender.com/st3ra1o (accessed on 9 December 2025).

Together, these observations underscore the need for a coordinated shift toward standardized, human-relevant experimentation and transparent reporting. Current evidence confirms that MNPs can trigger biological responses and cellular injury across multiple models; however, interpretation remains limited by inconsistent methodologies, unrealistic dosing, and the use of environmentally irrelevant particle types. Nevertheless, the observed cellular damage and inflammatory responses suggest a plausible link to chronic lung diseases, particularly with prolonged or repeated exposure (Figure 3). To advance the field, consensus on key experimental parameters is essential. Priority actions include (1) quantifying daily and annual human-relevant MNP exposure levels to inform risk assessment; (2) establishing standardized dose metrics and reporting practices, including harmonized units and reference ranges based on realistic exposure; (3) advocating for chronic, low-dose exposure studies that better reflect real-world scenarios; (4) employing physiologically relevant doses in in vitro, ex vivo, and in vivo studies, avoiding supraphysiological concentrations (e.g., >1000 μg/mL); (5) expanding beyond pristine polystyrene spheres to include diverse, environmentally representative polymers and weathered particles; (6) transitioning from simplified submerged cell cultures towards the adopting of advanced models such as organoids, air–liquid interface systems, and ex vivo platforms; and (7) developing an international consensus framework to guide experimental design, endpoint selection, and data transparency. Coordinated efforts in these areas are critical to generate robust, translatable evidence on pulmonary health risks associated with MNP exposure.

MNPs represent an emerging inhalation hazard with demonstrated capacity to disrupt pulmonary homeostasis through oxidative stress, immune activation, epithelial remodeling, and surfactant interference. While these effects are mechanistically linked to pathways underlying chronic lung disease, current evidence is largely derived from short-term, high-dose exposure studies. Bridging this gap with standardized, human-relevant experiments and realistic exposure scenarios is essential to clarify clinical significance and inform strategies for prevention and risk mitigation.

## Figures and Tables

**Table 1.** The three primary polymer compositions of micro- and nanoplastics commonly found in the environment (summarized and adapted from O’Brien et al.) [18].

Area	Micro- and Nanoplastic Composition
Outdoor air active sampling	PET, PE predominantly
Indoor air active sampling	Variable and site specific
Road dust deposition	PE, PVC, PP
Outdoor dust deposition	PET, PE, PA (nylon)
Indoor dust deposition	PET, PE, PP

PE: Polyethylene, PP: Polypropylene, PVC: Polyvinyl Chloride, PET: Polyethylene terephthalate, PA: Polyamide.

**Table 2.** Companies providing non-polystyrene micro- and nanoplastics suitable for research.

Company (Location)	Micro- and Nanoplastics Offered	Pricing	Website
Alfa Chemistry (New York, NY, USA)	Various plastics including PE, PP, PET, and more.	Pricing available on request	www.alfa-chemistry.com (accessed on 3 September 2025)
Cospheric (Somis, CA, USA)	PE, PP, PET, and other micro- and nanospheres (0.1–1000 μm)	Approximately USD 100–USD 300 per gram depending on type and size	www.cospheric.com (accessed on 3 September 2025)
Goodfellow Multiregional (Pittsburgh, PA, USA)	PE, PP, and biodegradable nylon plastic fibers (must be broken down into micro- and nanoplastics)	Starting from USD 110 for 1 m fiber and USD 170 for 100 g of granules. Varies by plastic type	www.goodfellow.com (accessed on 3 September 2025)
Polysciences, Inc. (Warrington, PA, USA)	PP, acrylic, and other polymer microparticles	Roughly USD 120–USD 220 per 100 g	www.polysciences.com (accessed on 3 September 2025)
Microplastic Solution (Toulouse, France)	PE, PP, PET, PVC fragments, fibers, and spheres (10–100 μm, 100–500 μm, 500–1000 μm)	Starting from USD 149.95–USD 399.95. Varies by polymer type and amount	www.microplasticsolution.com (accessed on 3 September 2025)
Lab261 (Palo Alto, CA, USA)	PE, PET, PVC, and more of various sizes	Pricing on request	www.lab261.com (accessed on 3 September 2025)

PE: Polyethylene, PP: Polypropylene, PVC: Polyvinyl Chloride, PET: Polyethylene terephthalate.

**Table 3.** In vitro studies with their associated outcomes.

Cell Type	Plastic Type, Size and Dose	Outcome Changes	Reference
Lung surfactant	Commercial and realistic PS-MPs, 1 μm, 0–10 μg/mL	Aged PS-MPs ↑ surface tension of lung surfactant ↑ Free radical formation	[151]
	PS-NPs, 600–800 nm, 0–1 mg/L	↑ Surface tension of lung surfactant ↑ Free radical formation	[152]
	PP-, PS- and PET-MPs and NPs (wide size range), 10, 100, and 1000 μg/mL	↑ Minimum surface tension and compressibility. Aggregation within the surfactant film	[109]
A549	PS-MPs, 1 and 10 μm, 0–100 μg/mL	↓ Cell division and cell metabolism Changes in cell morphology No change in cell viability	[122]
	Mixture of real MPs, 5–15 μm, 0–100 μg/mL	↓ Cell viability ↑ IL-6 and IL-8	[82]
	PS-NPs, 86–88 nm, 3.125–100 μg/mL	↑ Influenza A infection ↓ IFN-β and TANK-binding kinase 1	[158]
	PE-MPs and NPs < 10 μm, 0–1000 μg/mL	↑ ROS ↑ Cytokine production Slight ↓ viability (high dose)	[62]
	PS-NPs, 800 nm, 10–500 μg/mL	↓ Viability ↑ Hydrogen Peroxide ↑ Senescence and Apoptosis	[125]
	PET-NPs, 136 ± 10 nm, 0–125 μg/mL	↑ ROS ↑ DNA strand breaks No cytotoxicity	[63]
	PET-NPs, 122–296 nm, 0–197 μg/mL	↓ Viability ↓ Mitochondrial membrane potential ↑ ROS	[64]
	PS-NPs, 50 nm, 50 μg/mL	↑ Oxidative stress ↑ Cell death	[76]
	Aged PS-MPs, 1 and 5 μm, 0–20 μg/mL	↑ Toxicity with photoaging ↓ Cell number ↑ Division Arrest	[126]
	PTFE-MPs, 6–31 μm, up to 1000 μg/mL	↑ ROS No cytotoxicity	[65]
	Oxidized and virgin PS-MPs and NPs, 100 nm and 1 μm, 0–200 μg/mL	↑ ROS ↓ Mitochondrial membrane potential Oxidized more cytotoxic	[66]
	Aminated PS-NPs, 240 nm, up to 200 μg/mL	No effect on cytotoxicity, ROS, or DNA breaks	[159]
	PE-NPs, approximately 85 nm, up to 200 μg/mL	No cytotoxicity ↑ LDH and ↑ TNF-α	[124]
	PS-MPs, 1–5 μm, 0, 100, 250, 500 μg/mL, 2 days exposure	↑ TNF-α, ↑ caspase-9, BAX, BCL-2 ↓ Cell viability	[87]
	PE-MPs (6.5 μm to 1 mm) and PVC-MPs (6.5 μm to 25 μm), 0.5 and 5 μg/mL, 2 days exposure	↑ p21, p16, and Senescence-Associated β-Galactosidase ↑ TNF-α, IL-6, and IL-1β	[86]
	PS-MPs, 5 μm, 12.5–500 μg/mL, 24 h	↑ MDA, ↑ Long-chain-fatty-acid—CoA ligase 4, ↓ GPX4 ↓ cell viability	[134]
	PP-NPs, 0.66 ± 0.27 μm, 1000, 2000 or 4000 μg/mL	↓ ATP levels and mitochondrial membrane potential. ↑ TNF-α, IL-1β, IL-6, NF-κB ↓ Cell viability. All findings seen only at 4000 μg/mL	[77]
A549 and MRC-5 (fibroblast) cells	PS-MPs, 5 μm, 48 h, no dose mentioned	↑ β-catenin, α-SMA, vimentin, collagen when A549 cells are co cultured with MRC-5 cells	[147]
A549 and BEAS-2B	PE-MPs and NPs, 25–100 μg/mL; 200–9900 nm	↑ EMT markers ↑ Migration No invasion	[143]
	PS-MPs, 2 μm, 0.14 × 10^2^ to 5.68 × 10^5^ P/mL	↑ ROS only in differentiated A549 No change in viability	[68]
A549 and Calu-3	PS-Eu NPs, 279 nm, 0–100 μg/mL	↓ Surfactant proteins ↑ IL-6, ↑ TGF-β, ↑ Zonula Occludens-1, and ↑ Mucin-5B	[83]
A549 and THP-M co-culture	PS-NPs, 60 nm, 0–250 μg/mL	↑ ROS, oxidized/reduced glutathione ratio and MDA ↑ NLR family pyrin domain containing 3, MMP-9, Cle-Caspase-1, and IL-1β	[73]
BEAS-2B	Carboxylated, pristine and amino charged PS-NPs, 100 nm, 25–400 μg/mL	↓ Cell viability in amino charged PS ↑ ROS production	[69]
	PS-MPs, 1.72 μm, 1–1000 μg/cm^2^ (in vitro)	↓ Viability at high dose ↑ Heme oxygenase 1 and IL-6/8 ↓Zona Occludens-1 and ↓TEER (mitochondrial potential)	[84]
	PS-NPs, 80 nm, 100 μg/L	↑ ROS ↑ Mitochondrial damage	[70]
	PS-NPs, 100 nm, 60–220 μg/mL	↓ Mitochondrial membrane potential ↑ ROS, ↑ Ca^2+^, and ↑ cGAS/STING	[71]
	PET NPs, approximately 176 nm, 30-week exposure	↑ Genotoxicity (long-term only)	[132]
	PVC, PP, and PA NPs, <1–10 μm; PA < 1 μm at 19.6 μg/mL	↑ Cytotoxicity and IL-8 for PA NPs only	[91]
	LDPE-MPs and NPs, 147 nm–12.5 μm, up to 1000 μg/mL	↑ ROS EMT (↓ β-catenin, ↑ vimentin) ↓ basal and maximal respiration, ATP production, and spare respiratory capacity ↓ Cell division	[67]
	PS-MPs, 5 μm, 1, 5, 10, and 20 μg/mL	↓ Wound healing ↓ Cell viability ↑ ROS, ↑ TLR4, ↑ α-SMA, Extracellular matrix protein 1. ↓ FTH1, ↓ GPX4, and ↑ MDA	[74]
	Tire wear NPs, ~100 nm, 25, 50 and 100 μg/mL	↑ TGF-β, ↑ α-SMA, ↑ twinfilin-1	[110]
	PS-MPs and NPs, 10 μm or 20 nm; 50, 150 and 200 μg/mL	↓ Cell viability ↓ FTH1 and GPX4, ↑ MDA. All results in 20 nm group only	[140]
	PS-NPs, 100 nm and 200 nm, 0–400 μg/mL	↓ cell viability ↓ GPX4, ↑ MDA, FTH1 and Fe^2+^	[139]
BEAS-2B and Human Pulmonary Alveolar Epithelial Cells, and lung on chip	PS-NPs, 40 nm, 0–30 μg/cm^2^	↑ MDA, ↑ FTH1, ↓ reduced glutathione to oxidized glutathione ratio, ↓ GPX4 ↓ ATP production, Spare respiratory capacity, basal and maximal respiration ↓ Cell viability	[123]
TC-1	PS, PET, PVC, and PE- NPs, 100 nm, 0, 10, 20, 50, 100, and 200 μg/mL	↓ Cell viability ↑ MDA, ↓ SOD, GSH,	[120]
MLE12	PS-NPs, 100 nm, 0–400 μg/mL	↓ Cell viability ↑ p21, p16, and p27 ↑ IL-6, IL-8	[127]
Primary Human Nasal Epithelial Cells	PS-NPs, 50 and 500 nm, 100 μg/mL	No change in viability ↑ ROS ↓ Mitochondrial membrane potential	[72]
	PET-NPs, 10–250 nm, 0–100 ug/mL	↑ ROS ↓ Mitochondrial membrane potential	[75]
Human Lung Epithelial Cells	PET-MPs, ~45 μm dyed fibers, 2000 fibers	↓ Viability ↓ TEER ↑ CYP1A1/CYP1B1 (dyed only)	[21]
	PS-NPs, 40 nm, 7.5–30 μg/cm^2^	↓ Viability ↑ Apoptosis ↑ MMP-9 and Surfactant Protein A	[153]
Lung Organoids	Polyester, 0.2–0.4 mm fibers from dryer	Altered polarization	[24]
Lung Organoids (ALI)	Nylon MPs, 1–5 μm and 5–10 μm, 39 μg/mL	↓ Growth and epithelial differentiation	[20]
A549 and BEAS-2B (ALI)	PS, PVC, PP, PA- NPs, 50 nm–<1 μm, 1.39–112 μg/cm^2^ (in vitro)	No inflammatory changes No viability or cytokine changes	[90]
Primary Human Airway Epithelial Cells (ALI)	PS-NPs, 50 nm, up to 2500 μg/mL	↑ Macrophage inflammatory protein-1 ↓ IL-21, IL-15, IL-2, and TGF-β No cytotoxicity	[85]
Calu-3 (ALI)	PLA-NPs, 130 nm, 2.5, 10, and 20 μg/cm^2^, 1 day, 1 week (2.5 dose) and 2 weeks (2.5 dose)	↑ Cytokines/chemokines No ROS change ↑ Genotoxicity	[79]
Jurkat.EcoR cells	PS-NPs, 0.1 and 0.8 μm, 30–100 μg/mL	Phagocytosis via T-cell membrane protein 4 No change in ROS or cytokines	[102]
Primary human macrophages	PS/PMMA-MPs and NPs, 50 nm-1.1 μm, 1–100 μg/mL	↓ Pro-inflammatory markers, →M2 polarization	[105]
Primary Human macrophages and THP-1 cells	PS-MPs and NPs, 0.5–3 μm, 0–1500 μg/mL	↑ Metabolic activity, ↑ cytotoxicity for 0.5 μm only, ↑ Nitrite production	[104]
Primary murine macrophages	PS-MPs, 10 μm, 1 μg/mL	↑ Glycolysis, ↓ oxidative phosphorylation	[103]
NR8383 Alveolar macrophages	PS-MPs and NPs, 80 nm and 1 μm, 0–60 μg/mL	↓ Cell viability ↑ ROS production ↓ Mitochondrial membrane potential	[106]
THP-1	PS-NPs, 100 nm, 0–600 μg/mL	↑ MMP9 and MMP12 ↓ PTEN ↓ circRNA_SMG6	[107]
RAW 264.7 cells	PS-NPs, 90 nm, 0–200 μg/mL	↓ Cell viability ↑ LDH, ↑ MDA ↑ IL-1β, TNF-α, IL-6, and NLRP3	[108]
	PS-NH2, PS-COOH, and regular PS-MPs, 1 μm, 0–1000 μg/mL	↓ Cell viability in PS-NH_2_ group only	[78]

BALF: Bronchoalveolar lavage fluid, EMT: Epithelial–mesenchymal transition, FTH: Ferritin, GPX4: Glutathione peroxidase 4, GSH: Glutathione, IL: Interleukin, LDH: Lactate Dehydrogenase, MDA: Malondialdehyde, MIP: Macrophage inflammatory protein, MMP: Matrix metalloproteinase, MP: Microplastic, NP: Nanoplastic, PA: Polyamide, PE: Polyethylene, PET: Polyethylene terephthalate, PFT: Pulmonary function test, PP: Polypropylene, PS: Polystyrene, PVC: Polyvinyl Chloride, ROS: Reactive oxygen species, SMA: Smooth muscle actin, SOD: Superoxide Dismutase, TEER: Transepithelial electrical resistance, TGF: Transforming growth factor, TLR: Toll like receptor, TNF: Tumor necrosis Factor. ↑: content increase. ↓: content decrease.

**Table 4.** In vivo studies with their associated outcome changes.

Animal Model	Plastic Type, Size and Dose	Route of Administration	Outcome Changes	Reference
Mouse	PS-MPs, 1–5 and 10–20 μm, 40 mg/kg, 5×/week, 3 weeks	Intranasal	↑ Collagen deposition and fibrosis ↑ IL-8, TLR2, and NF-κB	[87]
	PS-MPs, 5 μm, 1.25 and 6.25 mg/kg, 3×/week, 3 weeks	Intratracheal	↑ Fibrosis ↑ IL-1β and TNF-α ↓ Surfactant production	[147]
	PVC-MPs, 6.5–25 μm, 25 mg/kg and 100 mg/kg, daily ×8 days	Intratracheal	↑ IL-1β, TNF-α, and White blood cell count ↑ Lung inflammation score ↓ Exercise capacity	[86]
	PS-MPs, 5 μm, 0.6–15 mg/kg, every 5 days for 60 days	Intratracheal	↑ Collagen deposition ↑ TLR4 ↓ GPX4 ↑ Fe^2+^ and MDA	[74]
	PP-MPs (6.4 μm), PS-MPs (21 μm), PE-MPs (17.53 μm), 5 mg/kg, daily ×2 weeks	Intratracheal	↑ Total cells (PS > others) ↑ Macs and neutrophils ↑ TLR2/4, IL-1β, NF-κB and NLRP3	[95]
	PE-NPs (225 nm), PS-NPs (52 nm), PET-NPs (265 nm), 2 mg/kg	Intranasal	↑ Surfactant disruption ↑ Pro-inflammatory cytokines PS had strongest effects	[109]
	PS-MPs, 5 μm, 0–2.0 mg/kg, daily ×4 weeks	Intranasal	↑ Lung fibrosis score ↑ MDA ↓ GSH and GPX4	[134]
	Tire wear NPs, ~100 nm, 0.125–1 mg/kg	Aerosol	↑ Collagen and fibrosis ↓ E-cadherin ↓ PFT values	[110]
	PS-NPs, 40 nm, 16–100 μg/day, 1 week-3 months	Inhalation	↑ Cytokines ↓ Lung function ↑ Lung fibrosis	[141]
	PS-NPs, 20 nm, 5–10 mg/kg, 8× in 15 days	Intratracheal	↑ Caspase-3, MDA, and ROS ↓GPX4 and Ferritin heavy chain	[140]
	PP-NPs, ~0.66 μm, 1–5 mg/kg, 5×/week, 4 weeks	Intratracheal	↑ Inflammatory cells and cytokines ↑ Foamy macrophage aggregates ↑ NF-κB	[77]
	PS-NPs, 100 and 200 nm, 12.5–25 mg/kg, 7 days	Intratracheal	↑ Fe^2+^, MDA ↑ Hypoxia-inducible factors-1α, and Heme oxygenase-1 ↓ GSH and GPX4	[139]
	PS, PET, PE, PVC-NPs, 100 nm, 1 mg/m^3^, daily ×2 weeks	Inhalation	↑ Macrophages, neutrophils, and cytokines in BALF ↑ Fe^2+^ ↓ GSH and Superoxide Dismutase PVC had strongest effect	[120]
	PS-NPs, 40 nm 100 μg/day, 4 weeks	Inhalation	↓ Mucin 1, ↓ GPX4, ferritin, and GSH, ↑ MDA ↑ MMP-9, Intercellular Adhesion Molecule 1, ↓ alpha-1 antitrypsin ↑ Monocyte chemoattractant protein-1, IL-6, TNF-α	[123]
	PS-MPs, 3 μm, 30 and 300 μg/mouse, every two weeks for total of 8 weeks	Intratracheal	↓ PFT values ↑ Pro-inflammatory cytokines ↑ TLR2/4 and NF-κB activation	[94]
	PS-MPs and NPs, 80 nm and 1 μm, 1.5 × 10^5^ particles/m^3^, 60 days	Inhalation	↑ Fibrosis ↓ E-cadherin, ↑ Vimentin, ↑ SNAIL-1, ↑ SNAIL-2 and ↑ TWIST (EMT markers) ↑ TNF-α, IL-1β, IL-6, IL-10 1 μm PS had greater effect than 80 nm	[106]
	PS-NPs, 0.4–0.6 μm, 4–16 μg/mL, 2 weeks	Inhalation	↑ Macs, total cells, and neutrophils in BALF ↑ Pro-inflammatory cytokines, ↑ Goblet cell hyperplasia, ↑ Fibrosis	[119]
	PS-NPs, 90 nm, 5 or 10 mg/kg, 3×/week ×6 weeks	Intranasal	↑ Pulmonary fibrosis ↑ Mitochondrial DNA release ↑ IL-1β, IL-18, NF-κB, and cGAS/STING	[108]
	PS-NPs, 100 nm, 5 mg/kg, 3× in 14 days	Intratracheal	↓ Weight ↑ NLRP3 and Caspase 1 ↑ TNF-α, IL-6 and IL-1β	[88]
Mouse (normal and asthmatic)	PE-MPs, 1–5 μm, 300 μg/day, 4 weeks	Intranasal	↑ Th2 response ↑ IL-33, ↑ Epithelial permeability ↑ Inflammatory cell infiltration	[154]
	Nylon MPs, 3 μm, 250 μg, days 1, 4, 7	Intranasal (with OVA challenge)	↑ IgE and inflammatory cytokines ↓ Epithelial barrier ↑ NLRP3	[155]
	PS-MPs and NPs, 213 and 2618 nm, 300 μg/mL, alternate days between days 21–27	Intranasal	↑ Pro-inflammatory cytokines ↑ Airway hyperreactivity ↑ BAX/BCL2 (NPs > MPs)	[156]
	PS, 300 μg, 8× in 24 days	Intranasal	↑ Macrophage aggregation ↑ Inflammation (greater in asthma)	[157]
Rat	PP-MPs, 1–10 μm, 0.8 and 4 mg/kg once then dissected at 3 days, 1 month and 3 months	Intratracheal and Intranasal	↑ Cytokine-induced neutrophil chemoattractant 1/2 and myeloperoxidase ↑ LDH ↑ Macrophage aggregates (at 3 months)	[89]
	PS-NPs, 100 nm, 0.5–2.0 mg/m^3^, 4 h/day, 5×/week, 35 days	Intraoral and Intranasal	↑ IL-6 and IL-8 ↑ p21 ↑ Airway hyperresponsiveness	[127]
	PS-NPs, 60 nm, 0–2 mg/m^3^, 90 days	intranasal	↑ TNF-α IL-1β, and NLRP3 ↑ MMP-9 ↑ MDA ↑ oxidized/reduced glutathione ratio	[73]
	PS-NPs, 100 nm; 0–1000 μg/mL, 48 h	Inhalation	↑ Airway hyperresponsiveness ↑ MMP9 and MMP12 ↓ circRNA_SMG6	[107]
	Amino-modified PS-MPs, 1 μm, 0.2 and 1.0 mg/kg, 3 days-3 months	Intratracheal	↑ ROS ↑ Cytokine-induced neutrophil chemoattractant 1/2 ↑ Total cell count (1 week)	[78]
	PS-NPs, 100 nm, 1 μm, or 2.5 μm; 0.5–2 mg, 3 days	Intratracheal	↑ TNF, IL-1β, IL-6, and IL-8 100 nm most toxic	[148]
	PS-NPs, 100 nm, 0.75–3.00 × 10^5^ particles/cm^3^, 2 weeks	Inhalation	↑ Respiratory rate ↓ Inspiratory time ↓ total serum White blood cells and lymphocytes ↑ TGF-β and TNF-α	[149]

BALF: Bronchoalveolar lavage fluid, EMT: Epithelial–mesenchymal transition, GPX4: Glutathione peroxidase 4, GSH: Glutathione, IL: Interleukin, LDH: Lactate dehydrogenase, MDA: Malondialdehyde, MMP: Matrix metalloproteinase, MNP: Micro/nanoplastic (umbrella term for microplastics and nanoplastics used when size is not specified), MP: Microplastic, NLRP3: NLR family pyrin domain containing 3, NP: Nanoplastic, PA: Polyamide, PE: Polyethylene, PET: Polyethylene terephthalate, PFT: Pulmonary function test, PP: Polypropylene, PS: Polystyrene, PVC: Polyvinyl Chloride, ROS: Reactive oxygen species, TGF: Transforming growth factor, TLR: Toll like receptor, TNF: Tumor necrosis Factor. ↑: content increase. ↓: content decrease.

## Data Availability

No new data were created or analyzed in this study.

## Abbreviations

The following abbreviations are used in this manuscript:

BALF: Bronchoalveolar Lavage Fluid
BW: Body Weight
CINC: Cytokine-induced neutrophil chemoattractant
CXCL: Chemokine Ligand
FTIR: Fourier-transform infrared
IL: Interleukin
GPX4: Glutathione peroxidase 4
FTH1: Ferritin heavy chain 1
MNP: Micro- and nanoplastic
MMP: Matrix Metalloproteinase
MP: Microplastic
NLRP3: Nod-like receptor family pyrin domain containing 3
NP: Nanoplastic
PE: Polyethylene
PET: Polyethylene terephthalate
PP: Polypropylene
PS: Polystyrene
PVC: Polyvinyl Chloride
ROS: Reactive Oxygen Species
TLR: Toll Like Receptor
TGF: Transforming Growth Factor
TNF: Tumor Necrosis Factor

## References

[1] Zuo L; He F; Sergakis GG; Koozehchian MS; Stimpfl JN; Rong Y; Diaz PT; Best TM Interrelated Role of Cigarette Smoking, Oxidative Stress, and Immune Response in COPD and Corresponding Treatments. Am. J. Physiol. Lung Cell. Mol. Physiol 2014, 307, L205–L218. 24879054 10.1152/ajplung.00330.2013

[2] Ling S Particulate Matter Air Pollution Exposure: Role in the Development and Exacerbation of Chronic Obstructive Pulmonary Disease. Int. J. Chron. Obstruct. Pulmon. Dis 2009, 233–243. 19554194 10.2147/copd.s5098 PMC2699820

[3] Chen Z; Liu N; Tang H; Gao X; Zhang Y; Kan H; Deng F; Zhao B; Zeng X; Sun Y; Health Effects of Exposure to Sulfur Dioxide, Nitrogen Dioxide, Ozone, and Carbon Monoxide between 1980 and 2019: A Systematic Review and Meta-analysis. Indoor Air 2022, 32, e13170. 36437665 10.1111/ina.13170

[4] Tang FR; Loke WK Sulfur Mustard and Respiratory Diseases. Crit. Rev. Toxicol 2012, 42, 688–702. 22742653 10.3109/10408444.2012.698405

[5] Ghabili K; Agutter PS; Ghanei M; Ansarin K; Shoja MM Mustard Gas Toxicity: The Acute and Chronic Pathological Effects. J. Appl. Toxicol 2010, 30, 627–643. 20836142 10.1002/jat.1581

[6] Mossman BT; Churg A Mechanisms in the Pathogenesis of Asbestosis and Silicosis. Am. J. Respir. Crit. Care Med 1998, 157, 1666–1680. 9603153 10.1164/ajrccm.157.5.9707141

[7] Wagner GR Asbestosis and Silicosis. Lancet 1997, 349, 1311–1315. 9142077 10.1016/S0140-6736(96)07336-9

[8] Stämpfli MR; Anderson GP How Cigarette Smoke Skews Immune Responses to Promote Infection, Lung Disease and Cancer. Nat. Rev. Immunol 2009, 9, 377–384. 19330016 10.1038/nri2530

[9] US EPA. Microplastics Research. Available online: https://www.epa.gov/water-research/microplastics-research (accessed on 3 April 2025).

[10] Thompson RC; Olsen Y; Mitchell RP; Davis A; Rowland SJ; John AWG; McGonigle D; Russell AE Lost at Sea: Where Is All the Plastic? Science 2004, 304, 838. 15131299 10.1126/science.1094559

[11] Fang C; Awoyemi OS; Saianand G; Xu L; Niu J; Naidu R Characterising Microplastics in Indoor Air: Insights from Raman Imaging Analysis of Air Filter Samples. J. Hazard. Mater 2024, 464, 132969. 37956564 10.1016/j.jhazmat.2023.132969

[12] Young N Microplastics Are in the Air We Breathe and in Earth’s Atmosphere, and They Affect the Climate. Available online: https://www.greenpeace.org/aotearoa/story/microplastics-are-in-the-air-we-breathe-and-in-earths-atmosphere-and-they-affect-the-climate/ (accessed on 28 March 2025).

[13] Torres-Agullo A; Karanasiou A; Moreno T; Lacorte S Airborne Microplastic Particle Concentrations and Characterization in Indoor Urban Microenvironments. Environ. Pollut 2022, 308, 119707. 35803441 10.1016/j.envpol.2022.119707

[14] Vega-Herrera A; Garcia-Torné M; Borrell-Diaz X; Abad E; Llorca M; Villanueva CM; Farré M Exposure to Micro(Nano)Plastics Polymers in Water Stored in Single-Use Plastic Bottles. Chemosphere 2023, 343, 140106. 37689148 10.1016/j.chemosphere.2023.140106

[15] World Health Organization. Dietary and Inhalation Exposure to Nano- and Microplastic Particles and Potential Implications for Human Health; World Health Organization: Geneva, Switzerland, 2022; p. 154.

[16] Liao Z; Ji X; Ma Y; Lv B; Huang W; Zhu X; Fang M; Wang Q; Wang X; Dahlgren R; Airborne Microplastics in Indoor and Outdoor Environments of a Coastal City in Eastern China. J. Hazard. Mater 2021, 417, 126007. 33992007 10.1016/j.jhazmat.2021.126007

[17] Amato-Lourenço LF; Carvalho-Oliveira R; Júnior GR; dos Santos Galvão L; Ando RA; Mauad T Presence of Airborne Microplastics in Human Lung Tissue. J. Hazard. Mater 2021, 416, 126124. 34492918 10.1016/j.jhazmat.2021.126124

[18] O’Brien S; Rauert C; Ribeiro F; Okoffo ED; Burrows SD; O’Brien JW; Wang X; Wright SL; Thomas KV There’s Something in the Air: A Review of Sources, Prevalence and Behaviour of Microplastics in the Atmosphere. Sci. Total Environ 2023, 874, 162193. 36828069 10.1016/j.scitotenv.2023.162193

[19] Cole M A Novel Method for Preparing Microplastic Fibers. Sci. Rep 2016, 6, 34519. 27694820 10.1038/srep34519 PMC5046121

[20] Song S; van Dijk F; Vasse GF; Liu Q; Gosselink IF; Weltjens E; Remels AHV; de Jager MH; Bos S; Li C; Inhalable Textile Microplastic Fibers Impair Airway Epithelial Differentiation. Am. J. Respir. Crit. Care Med 2024, 209, 427–443. 37971785 10.1164/rccm.202211-2099OC

[21] O’Connor A; Villalobos Santeli A; Nannu Shankar S; Shirkhani A; Baker TR; Wu C-Y; Mehrad B; Ferguson PL; Sabo-Attwood T Toxicity of Microplastic Fibers Containing Azobenzene Disperse Dyes to Human Lung Epithelial Cells Cultured at an Air-Liquid Interface. J. Hazard. Mater 2024, 480, 136280. 39515142 10.1016/j.jhazmat.2024.136280 PMC11698483

[22] Paplińska-Goryca M; Misiukiewicz-Stępień P; Wróbel M; Mycroft-Rzeszotarska K; Adamska D; Rachowka J; Królikowska M; Goryca K; Krenke R The Impaired Response of Nasal Epithelial Cells to Microplastic Stimulation in Asthma and COPD. Sci. Rep 2025, 15, 4242. 39905077 10.1038/s41598-025-87242-x PMC11794662

[23] Bardawil CE; Dobbins J; Lankford S; Chowdrey S; Shumway J; Balamayooran G; Schaack C; Dhupar R Making Fluorescent Nylon, Polypropylene, and Polystyrene Microplastics for In Vivo and In Vitro Imaging. Microplastics 2025, 4, 84. 10.3390/microplastics4040084 PMC13245631 42266572

[24] Winkler AS; Cherubini A; Rusconi F; Santo N; Madaschi L; Pistoni C; Moschetti G; Sarnicola ML; Crosti M; Rosso L; Human Airway Organoids and Microplastic Fibers: A New Exposure Model for Emerging Contaminants. Environ. Int 2022, 163, 107200. 35349910 10.1016/j.envint.2022.107200

[25] Parker LA; Höppener EM; van Amelrooij EF; Henke S; Kooter IM; Grigoriadi K; Nooijens MGA; Brunner AM; Boersma A Protocol for the Production of Micro- and Nanoplastic Test Materials. Microplastics Nanoplastics 2023, 3, 10.

[26] Lievens S; Vervoort E; Poma G; Covaci A; Van Der Borght M A Production and Fractionation Protocol for Polyvinyl Chloride Microplastics. Methods Protoc. 2023, 6, 15. 36827502 10.3390/mps6010015 PMC9962165

[27] Tanaka K; Takahashi Y; Kuramochi H; Osako M; Tanaka S; Suzuki G Preparation of Nanoscale Particles of Five Major Polymers as Potential Standards for the Study of Nanoplastics. Small 2021, 17, 2105781. 10.1002/smll.202105781 34719868

[28] Rodríguez-Hernández AG; Muñoz-Tabares JA; Aguilar-Guzmán JC; Vazquez-Duhalt R A Novel and Simple Method for Polyethylene Terephthalate (PET) Nanoparticle Production. Environ. Sci. Nano 2019, 6, 2031–2036.

[29] Balakrishnan G; Déniel M; Nicolai T; Chassenieux C; Lagarde F Towards More Realistic Reference Microplastics and Nanoplastics: Preparation of Polyethylene Micro/Nanoparticles with a Biosurfactant. Environ. Sci. Nano 2019, 6, 315–324.

[30] Mariano S; Tacconi S; Fidaleo M; Rossi M; Dini L Micro and Nanoplastics Identification: Classic Methods and Innovative Detection Techniques. Front. Toxicol 2021, 3, 636640. 35295124 10.3389/ftox.2021.636640 PMC8915801

[31] Hidalgo-Ruz V; Gutow L; Thompson RC; Thiel M Microplastics in the Marine Environment: A Review of the Methods Used for Identification and Quantification. Environ. Sci. Technol 2012, 46, 3060–3075. 22321064 10.1021/es2031505

[32] Huang Z; Hu B; Wang H Analytical Methods for Microplastics in the Environment: A Review. Environ. Chem. Lett 2023, 21, 383–401. 36196263 10.1007/s10311-022-01525-7 PMC9521859

[33] Prata JC; da Costa JP; Duarte AC; Rocha-Santos T Methods for Sampling and Detection of Microplastics in Water and Sediment: A Critical Review. TrAC Trends Anal. Chem 2019, 110, 150–159.

[34] Schwaferts C; Niessner R; Elsner M; Ivleva NP Methods for the Analysis of Submicrometer- and Nanoplastic Particles in the Environment. TrAC Trends Anal. Chem 2019, 112, 52–65.

[35] Käppler A; Fischer D; Oberbeckmann S; Schernewski G; Labrenz M; Eichhorn K-J; Voit B Analysis of Environmental Microplastics by Vibrational Microspectroscopy: FTIR, Raman or Both? Anal. Bioanal. Chem 2016, 408, 8377–8391. 27722940 10.1007/s00216-016-9956-3

[36] Rozman U; Turk T; Skalar T; Zupančič M; Čelan Korošin N; Marinšek M; Olivero-Verbel J; Kalčíková G An Extensive Characterization of Various Environmentally Relevant Microplastics—Material Properties, Leaching and Ecotoxicity Testing. Sci. Total Environ 2021, 773, 145576. 33940734 10.1016/j.scitotenv.2021.145576

[37] Müller YK; Wernicke T; Pittroff M; Witzig CS; Storck FR; Klinger J; Zumbülte N Microplastic Analysis—Are We Measuring the Same? Results on the First Global Comparative Study for Microplastic Analysis in a Water Sample. Anal. Bioanal. Chem 2020, 412, 555–560. 31848670 10.1007/s00216-019-02311-1

[38] Jenner LC; Rotchell JM; Bennett RT; Cowen M; Tentzeris V; Sadofsky LR Detection of Microplastics in Human Lung Tissue Using μFTIR Spectroscopy. Sci. Total Environ 2022, 831, 154907. 35364151 10.1016/j.scitotenv.2022.154907

[39] Xu J-L; Thomas KV; Luo Z; Gowen AA FTIR and Raman Imaging for Microplastics Analysis: State of the Art, Challenges and Prospects. TrAC Trends Anal. Chem 2019, 119, 115629.

[40] Ivleva NP; Wiesheu AC; Niessner R Microplastic in Aquatic Ecosystems. Angew. Chem. Int. Ed 2017, 56, 1720–1739. 10.1002/anie.201606957 27618688

[41] Weber F; Zinnen A; Kerpen J Development of a Machine Learning-Based Method for the Analysis of Microplastics in Environmental Samples Using μ-Raman Spectroscopy. Microplastics Nanoplastics 2023, 3, 9.

[42] Shim WJ; Hong SH; Eo SE Identification Methods in Microplastic Analysis: A Review. Anal. Methods 2017, 9, 1384–1391.

[43] Wang Z-M; Wagner J; Ghosal S; Bedi G; Wall S SEM/EDS and Optical Microscopy Analyses of Microplastics in Ocean Trawl and Fish Guts. Sci. Total Environ 2017, 603–604, 616–626. 10.1016/j.scitotenv.2017.06.047 28646780

[44] Dawson AL; Kawaguchi S; King CK; Townsend KA; King R; Huston WM; Bengtson Nash SM Turning Microplastics into Nanoplastics through Digestive Fragmentation by Antarctic Krill. Nat. Commun 2018, 9, 1001. 29520086 10.1038/s41467-018-03465-9 PMC5843626

[45] Shim WJ; Song YK; Hong SH; Jang M Identification and Quantification of Microplastics Using Nile Red Staining. Mar. Pollut. Bull 2016, 113, 469–476. 28340965 10.1016/j.marpolbul.2016.10.049

[46] Huang X; Saha SC; Saha G; Francis I; Luo Z Transport and Deposition of Microplastics and Nanoplastics in the Human Respiratory Tract. Environ. Adv 2024, 16, 100525.

[47] Triantafyllaki M; Chalvatzaki E; Torres-Agullo A; Karanasiou A; Lacorte S; Drossinos Y; Lazaridis M The Fate of Airborne Microfibers in the Human Respiratory Tract in Different Microenvironments. Sci. Total Environ 2024, 953, 176000. 39233080 10.1016/j.scitotenv.2024.176000

[48] Huang S; Huang X; Bi R; Guo Q; Yu X; Zeng Q; Huang Z; Liu T; Wu H; Chen Y; Detection and Analysis of Microplastics in Human Sputum. Environ. Sci. Technol 2022, 56, 2476–2486. 35073488 10.1021/acs.est.1c03859

[49] Qiu L; Lu W; Tu C; Li X; Zhang H; Wang S; Chen M; Zheng X; Wang Z; Lin M; Evidence of Microplastics in Bronchoalveolar Lavage Fluid among Never-Smokers: A Prospective Case Series. Environ. Sci. Technol 2023, 57, 2435–2444. 36718593 10.1021/acs.est.2c06880

[50] Lu W; Li X; Wang S; Tu C; Qiu L; Zhang H; Zhong C; Li S; Liu Y; Liu J; New Evidence of Microplastics in the Lower Respiratory Tract: Inhalation through Smoking. Environ. Sci. Technol 2023, 57, 8496–8505. 37267095 10.1021/acs.est.3c00716

[51] Zhu L; Kang Y; Ma M; Wu Z; Zhang L; Hu R; Xu Q; Zhu J; Gu X; An L Tissue Accumulation of Microplastics and Potential Health Risks in Human. Sci. Total Environ 2024, 915, 170004. 38220018 10.1016/j.scitotenv.2024.170004

[52] Baeza-Martínez C; Olmos S; González-Pleiter M; López-Castellanos J; García-Pachón E; Masiá-Canuto M; Hernández-Blasco L; Bayo J First Evidence of Microplastics Isolated in European Citizens’ Lower Airway. J. Hazard. Mater 2022, 438, 129439. 35777146 10.1016/j.jhazmat.2022.129439

[53] Pezzulo AA; Starner TD; Scheetz TE; Traver GL; Tilley AE; Harvey B-G; Crystal RG; McCray PB; Zabner J The Air-Liquid Interface and Use of Primary Cell Cultures Are Important to Recapitulate the Transcriptional Profile of in Vivo Airway Epithelia. Am. J. Physiol. Lung Cell. Mol. Physiol 2011, 300, L25–L31. 20971803 10.1152/ajplung.00256.2010 PMC3023285

[54] He R-W; Braakhuis HM; Vandebriel RJ; Staal YCM; Gremmer ER; Fokkens PHB; Kemp C; Vermeulen J; Westerink RHS; Cassee FR Optimization of an Air-Liquid Interface in Vitro Cell Co-Culture Model to Estimate the Hazard of Aerosol Exposures. J. Aerosol Sci 2021, 153, 105703. 33658726 10.1016/j.jaerosci.2020.105703 PMC7874005

[55] Upadhyay S; Palmberg L Air-Liquid Interface: Relevant In Vitro Models for Investigating Air Pollutant-Induced Pulmonary Toxicity. Toxicol. Sci 2018, 164, 21–30. 29534242 10.1093/toxsci/kfy053

[56] Issa R; Lozano N; Kostarelos K; Vranic S Functioning Human Lung Organoids Model Pulmonary Tissue Response from Carbon Nanomaterial Exposures. Nano Today 2024, 56, 102254.

[57] Lee J; Baek H; Jang J; Park J; Cha S-R; Hong S-H; Kim J; Lee J-H; Hong I-S; Wang S-J; Establishment of a Human Induced Pluripotent Stem Cell Derived Alveolar Organoid for Toxicity Assessment. Toxicol. Vitro 2023, 89, 105585. 10.1016/j.tiv.2023.105585 36931533

[58] Sengupta A; Schmid S; Grangier N; Dorn A; Hebestreit M; Hugi A; Žajdlíková K; Herbst A; Losada-Oliva P; Ortolf-Wahl H; A Next-Generation System for Smoke Inhalation Integrated with a Breathing Lung-on-Chip to Model Human Lung Responses to Cigarette Exposure. Sci. Rep 2025, 15, 18181. 40414911 10.1038/s41598-025-00438-z PMC12104466

[59] Yang S; Zhang T; Ge Y; Cheng Y; Yin L; Pu Y; Chen Z; Liang G Sentinel Supervised Lung-on-a-Chip: A New Environmental Toxicology Platform for Nanoplastic-Induced Lung Injury. J. Hazard. Mater 2023, 458, 131962. 37406524 10.1016/j.jhazmat.2023.131962

[60] Stucki AO; Sauer UG; Allen DG; Kleinstreuer NC; Perron MM; Yozzo KL; Lowit AB; Clippinger AJ Differences in the Anatomy and Physiology of the Human and Rat Respiratory Tracts and Impact on Toxicological Assessments. Regul. Toxicol. Pharmacol 2024, 150, 105648. 38772524 10.1016/j.yrtph.2024.105648 PMC11198871

[61] Driscoll KE; Costa DL; Hatch G; Henderson R; Oberdorster G; Salem H; Schlesinger RB Intratracheal Instillation as an Exposure Technique for the Evaluation of Respiratory Tract Toxicity: Uses and Limitations. Toxicol. Sci 2000, 55, 24–35. 10788556 10.1093/toxsci/55.1.24

[62] Gautam R; Jo JH; Acharya M; Maharjan A; Lee DE; Pramod PB; Kim CY; Kim KS; Kim HA; Heo Y Evaluation of Potential Toxicity of Polyethylene Microplastics on Human Derived Cell Lines. Sci. Total Environ 2022, 838, 156089. 35605862 10.1016/j.scitotenv.2022.156089

[63] Alzaben M; Burve R; Loeschner K; Møller P; Roursgaard M Nanoplastics from Ground Polyethylene Terephthalate Food Containers: Genotoxicity in Human Lung Epithelial A549 Cells. Mutat. Res. Toxicol. Environ. Mutagen 2023, 892, 503705. 10.1016/j.mrgentox.2023.503705 37973296

[64] Zhang H; Zhang S; Duan Z; Wang L Pulmonary Toxicology Assessment of Polyethylene Terephthalate Nanoplastic Particles in Vitro. Environ. Int 2022, 162, 107177. 35303532 10.1016/j.envint.2022.107177

[65] Pramod BKC; Maharjan A; Acharya M; Lee D; Kusma S; Gautam R; Kwon J-T; Kim C; Kim K; Kim H; Polytetrafluorethylene Microplastic Particles Mediated Oxidative Stress, Inflammation, and Intracellular Signaling Pathway Alteration in Human Derived Cell Lines. Sci. Total Environ 2023, 897, 165295. 37419366 10.1016/j.scitotenv.2023.165295

[66] Laganà A; Visalli G; Facciolà A; Celesti C; Iannazzo D; Di Pietro A Uptake of Breathable Nano- and Micro-Sized Polystyrene Particles: Comparison of Virgin and Oxidised nPS/mPS in Human Alveolar Cells. Toxics 2023, 11, 686. 37624191 10.3390/toxics11080686 PMC10459673

[67] Vailionytė A; Uogintė I; Pajarskienė J; Bagdonas E; Jelinskas T; Ignatjev I; Byčenkienė S; Aldonytė R In Vitro Effects of Aged Low-Density Polyethylene Micro(Nano)Plastic Particles on Human Airway Epithelial Cells. Environ. Pollut 2025, 374, 126186. 40185180 10.1016/j.envpol.2025.126186

[68] Peng M; Vercauteren M; Grootaert C; Rajkovic A; Boon N; Janssen C; Asselman J Cellular and Bioenergetic Effects of Polystyrene Microplastic in Function of Cell Type, Differentiation Status and Post-Exposure Time. Environ. Pollut 2023, 337, 122550. 37716692 10.1016/j.envpol.2023.122550

[69] Jeon MS; Kim JW; Han YB; Jeong MH; Kim HR; Sik Kim H; Park YJ; Chung KH Polystyrene Microplastic Particles Induce Autophagic Cell Death in BEAS-2B Human Bronchial Epithelial Cells. Environ. Toxicol 2023, 38, 359–367. 36485005 10.1002/tox.23705

[70] Han M; Liang J; Wang K; Si Q; Zhu C; Zhao Y; Khan NAK; Abdullah ALB; Shau-Hwai AT; Li YM; Integrin A5B1-Mediated Endocytosis of Polystyrene Nanoplastics: Implications for Human Lung Disease and Therapeutic Targets. Sci. Total Environ 2024, 953, 176017. 39236815 10.1016/j.scitotenv.2024.176017

[71] Xuan L; Wang Y; Qu C; Yan Y; Yi W; Yang J; Skonieczna M; Chen C; Miszczyk J; Ivanov DS; Metabolomics Reveals That PS-NPs Promote Lung Injury by Regulating Prostaglandin B1 through the cGAS-STING Pathway. Chemosphere 2023, 342, 140108. 37714480 10.1016/j.chemosphere.2023.140108

[72] Annangi B; Villacorta A; López-Mesas M; Fuentes-Cebrian V; Marcos R; Hernández A Hazard Assessment of Polystyrene Nanoplastics in Primary Human Nasal Epithelial Cells, Focusing on the Autophagic Effects. Biomolecules 2023, 13, 220. 36830590 10.3390/biom13020220 PMC9953511

[73] Bu N; Du Q; Xiao T; Jiang Z; Lin J; Chen W; Fan B; Wang J; Xia H; Cheng C; Mechanism of S-Palmitoylation in Polystyrene Nanoplastics-Induced Macrophage Cuproptosis Contributing to Emphysema through Alveolar Epithelial Cell Pyroptosis. ACS Nano 2025, 19, 18708–18728. 40335889 10.1021/acsnano.5c02892

[74] Kang H; Huang D; Zhang W; Wang JY; Liu Z; Wang Z; Jiang G; Gao A Inhaled Polystyrene Microplastics Impaired Lung Function through Pulmonary Flora/TLR4-Mediated Iron Homeostasis Imbalance. Sci. Total Environ 2024, 946, 174300. 38936707 10.1016/j.scitotenv.2024.174300

[75] Annangi B; Villacorta A; Vela L; Tavakolpournegari A; Marcos R; Hernández A Effects of True-to-Life PET Nanoplastics Using Primary Human Nasal Epithelial Cells. Environ. Toxicol. Pharmacol 2023, 100, 104140. 37137422 10.1016/j.etap.2023.104140

[76] Zhao Y; Fan W-T; Jin K-Q; Yan J; Qi Y-T; Huang W-H; Liu Y-L Real-Time Quantification of Nanoplastics-Induced Oxidative Stress in Stretching Alveolar Cells. ACS Nano 2024, 18, 6176–6185. 38359155 10.1021/acsnano.3c08851

[77] Woo JH; Seo HJ; Lee JY; Lee I; Jeon K; Kim B; Lee K Polypropylene Nanoplastic Exposure Leads to Lung Inflammation through P38-Mediated NF-κB Pathway Due to Mitochondrial Damage. Part. Fibre Toxicol 2023, 20, 2. 36624477 10.1186/s12989-022-00512-8 PMC9829531

[78] Tomonaga T; Higashi H; Izumi H; Nishida C; Sato K; Nakamura Y; Morimoto T; Higashi Y; Kojima T; Sakurai K; Comparison of Lung Disorders Following Intratracheal Instillation of Polystyrene Microplastics with Different Surface Functional Groups. J. Occup. Health 2025, 67, uiaf006. 39898983 10.1093/joccuh/uiaf006 PMC11894927

[79] García-Rodríguez A; Gutiérrez J; Villacorta A; Arribas Arranz J; Romero-Andrada I; Lacoma A; Marcos R; Hernández A; Rubio L Polylactic Acid Nanoplastics (PLA-NPLs) Induce Adverse Effects on an in Vitro Model of the Human Lung Epithelium: The Calu-3 Air-Liquid Interface (ALI) Barrier. J. Hazard. Mater 2024, 475, 134900. 38878440 10.1016/j.jhazmat.2024.134900

[80] Azad N; Rojanasakul Y; Vallyathan V Inflammation and Lung Cancer: Roles of Reactive Oxygen/Nitrogen Species. J. Toxicol. Environ. Health Part B 2008, 11, 1–15. 10.1080/10937400701436460 18176884

[81] Chabot F; Mitchell JA; Gutteridge JM; Evans TW Reactive Oxygen Species in Acute Lung Injury. Eur. Respir. J 1998, 11, 745–757. 9596132

[82] Bengalli R; Zerboni A; Bonfanti P; Saibene M; Mehn D; Cella C; Ponti J; La Spina R; Mantecca P Characterization of Microparticles Derived from Waste Plastics and Their Bio-Interaction with Human Lung A549 Cells. J. Appl. Toxicol 2022, 42, 2030–2044. 35929361 10.1002/jat.4372 PMC9805234

[83] Michelini S; Mawas S; Kurešepi E; Barbero F; Šimunović K; Miremont D; Devineau S; Schicht M; Ganin V; Haugen ØP; Pulmonary Hazards of Nanoplastic Particles: A Study Using Polystyrene in in Vitro Models of the Alveolar and Bronchial Epithelium. J. Nanobiotechnol 2025, 23, 388. 10.1186/s12951-025-03419-6 PMC12117733 40426130

[84] Dong C-D; Chen C-W; Chen Y-C; Chen H-H; Lee J-S; Lin C-H Polystyrene Microplastic Particles: In Vitro Pulmonary Toxicity Assessment. J. Hazard. Mater 2020, 385, 121575. 31727530 10.1016/j.jhazmat.2019.121575

[85] Breidenbach JD; French BW; Shrestha U; Adya ZK; Wooten RM; Fribley AM; Malhotra D; Haller ST; Kennedy DJ Acute Exposure to Aerosolized Nanoplastics Modulates Redox-Linked Immune Responses in Human Airway Epithelium. Antioxidants 2025, 14, 424. 40298680 10.3390/antiox14040424 PMC12024294

[86] Jin W; Zhang W; Tang H; Wang P; Zhang Y; Liu S; Qiu J; Chen H; Wang L; Wang R; Microplastics Exposure Causes the Senescence of Human Lung Epithelial Cells and Mouse Lungs by Inducing ROS Signaling. Environ. Int 2024, 185, 108489. 38367553 10.1016/j.envint.2024.108489

[87] Cao J; Xu R; Geng Y; Xu S; Guo M Exposure to Polystyrene Microplastics Triggers Lung Injury via Targeting Toll-like Receptor 2 and Activation of the NF-κB Signal in Mice. Environ. Pollut 2023, 320, 121068. 36641069 10.1016/j.envpol.2023.121068

[88] Wu Y; Yao Y; Bai H; Shimizu K; Li R; Zhang C Investigation of Pulmonary Toxicity Evaluation on Mice Exposed to Polystyrene Nanoplastics: The Potential Protective Role of the Antioxidant N-Acetylcysteine. Sci. Total Environ 2023, 855, 158851. 36155047 10.1016/j.scitotenv.2022.158851

[89] Tomonaga T; Higashi H; Izumi H; Nishida C; Kawai N; Sato K; Morimoto T; Higashi Y; Yatera K; Morimoto Y Investigation of Pulmonary Inflammatory Responses Following Intratracheal Instillation of and Inhalation Exposure to Polypropylene Microplastics. Part. Fibre Toxicol 2024, 21, 29. 39107780 10.1186/s12989-024-00592-8 PMC11301944

[90] Gosselink IF; van Schooten FJ; Drittij MJ; Höppener EM; Leonhardt P; Moschini E; Serchi T; Gutleb AC; Kooter IM; Remels AH Assessing Toxicity of Amorphous Nanoplastics in Airway- and Lung Epithelial Cells Using Air-Liquid Interface Models. Chemosphere 2024, 368, 143702. 39522701 10.1016/j.chemosphere.2024.143702

[91] Gosselink IF; Leonhardt P; Höppener EM; Smelt R; Drittij MJ; Davigo M; van den Akker GGH; Kooter IM; Welting TJM; van Schooten FJ; Size- and Polymer-Dependent Toxicity of Amorphous Environmentally Relevant Micro- and Nanoplastics in Human Bronchial Epithelial Cells. Microplast. Nanoplast 2025, 5, 19. 40385552 10.1186/s43591-025-00126-9 PMC12081513

[92] Takeda K; Kaisho T; Akira S Toll-Like Receptors. Annu. Rev. Immunol 2003, 21, 335–376. 12524386 10.1146/annurev.immunol.21.120601.141126

[93] Jiang D; Liang J; Li Y; Noble PW The Role of Toll-like Receptors in Non-Infectious Lung Injury. Cell Res. 2006, 16, 693–701. 16894359 10.1038/sj.cr.7310085

[94] Xia Q; Wei Y; Hu L; Zeng F; Chen Y; Xu D; Sun Y; Zhao L; Li Y; Pang G; Inhalation of Microplastics Induces Inflammatory Injuries in Multiple Murine Organs via the Toll-like Receptor Pathway. Environ. Sci. Technol 2024, 58, 18603–18618. 39389766 10.1021/acs.est.4c06637

[95] Kwabena Danso I; Woo JH; Hoon Baek S; Kim K; Lee K Pulmonary Toxicity Assessment of Polypropylene, Polystyrene, and Polyethylene Microplastic Fragments in Mice. Toxicol. Res 2024, 40, 313–323. 38525136 10.1007/s43188-023-00224-x PMC10959865

[96] Inoue K; Takano H; Yanagisawa R; Hirano S; Ichinose T; Shimada A; Yoshikawa T The Role of Toll-like Receptor 4 in Airway Inflammation Induced by Diesel Exhaust Particles. Arch. Toxicol 2006, 80, 275–279. 16254717 10.1007/s00204-005-0040-6

[97] Shoenfelt J; Mitkus RJ; Zeisler R; Spatz RO; Powell J; Fenton MJ; Squibb KA; Medvedev AE Involvement of TLR2 and TLR4 in Inflammatory Immune Responses Induced by Fine and Coarse Ambient Air Particulate Matter. J. Leukoc. Biol 2009, 86, 303–312. 19406832 10.1189/jlb.1008587 PMC2726765

[98] Fels AO; Cohn ZA The Alveolar Macrophage. J. Appl. Physiol 1986, 60, 353–369. 3005225 10.1152/jappl.1986.60.2.353

[99] Champion JA; Walker A; Mitragotri S Role of Particle Size in Phagocytosis of Polymeric Microspheres. Pharm. Res 2008, 25, 1815–1821. 18373181 10.1007/s11095-008-9562-y PMC2793372

[100] Doshi N; Mitragotri S Macrophages Recognize Size and Shape of Their Targets. PLoS ONE 2010, 5, e10051. 20386614 10.1371/journal.pone.0010051 PMC2850372

[101] Baranov MV; Kumar M; Sacanna S; Thutupalli S; van den Bogaart G Modulation of Immune Responses by Particle Size and Shape. Front. Immunol 2021, 11, 607945. 33679696 10.3389/fimmu.2020.607945 PMC7927956

[102] Kuroiwa M; Yamaguchi SI; Kato Y; Hori A; Toyoura S; Nakahara M; Morimoto N; Nakayama M Tim4, a Macrophage Receptor for Apoptotic Cells, Binds Polystyrene Microplastics via Aromatic-Aromatic Interactions. Sci. Total Environ 2023, 875, 162586. 36871719 10.1016/j.scitotenv.2023.162586

[103] Merkley SD; Moss HC; Goodfellow SM; Ling CL; Meyer-Hagen JL; Weaver J; Campen MJ; Castillo EF Polystyrene Microplastics Induce an Immunometabolic Active State in Macrophages. Cell Biol. Toxicol 2022, 38, 31–41. 34021430 10.1007/s10565-021-09616-x PMC8606615

[104] Adler MY; Issoual I; Rückert M; Deloch L; Meier C; Tschernig T; Alexiou C; Pfister F; Ramsperger AF; Laforsch C; Effect of Micro- and Nanoplastic Particles on Human Macrophages. J. Hazard. Mater 2024, 471, 134253. 38642497 10.1016/j.jhazmat.2024.134253

[105] Wolff CM; Singer D; Schmidt A; Bekeschus S Immune and Inflammatory Responses of Human Macrophages, Dendritic Cells, and T-Cells in Presence of Micro- and Nanoplastic of Different Types and Sizes. J. Hazard. Mater 2023, 459, 132194. 37572607 10.1016/j.jhazmat.2023.132194

[106] Chen L; Liu Y; Li H; Lin S; Wang X; Fang J; Diao X; Wang L; Yang Z; Cai Z Size-Dependent Pulmonary Toxicity and Whole-Body Distribution of Inhaled Micro/Nanoplastic Particles in Male Mice from Chronic Exposure. Environ. Sci. Technol 2025, 59, 6993–7003. 40181497 10.1021/acs.est.4c14232

[107] Sun X; Xiao T; Qin J; Song Y; Lu K; Ding R; Shi W; Bian Q Mechanism of circRNA_SMG6 Mediating Lung Macrophage ECM Degradation via miR-570–3p in Microplastics-Induced Emphysema. Environ. Int 2024, 187, 108701. 38685156 10.1016/j.envint.2024.108701

[108] Liu J; Xu F; Guo M; Gao D; Song Y Nasal Instillation of Polystyrene Nanoplastics Induce Lung Injury via Mitochondrial DNA Release and Activation of the Cyclic GMP-AMP Synthase-Stimulator of Interferon Genes-Signaling Cascade. Sci. Total Environ 2024, 948, 174674. 39002594 10.1016/j.scitotenv.2024.174674

[109] Xu X; Goros RA; Dong Z; Meng X; Li G; Chen W; Liu S; Ma J; Zuo YY Microplastics and Nanoplastics Impair the Biophysical Function of Pulmonary Surfactant by Forming Heteroaggregates at the Alveolar-Capillary Interface. Environ. Sci. Technol 2023, 57, 21050–21060. 38055865 10.1021/acs.est.3c06668

[110] Li Y; Shi T; Li X; Sun H; Xia X; Ji X; Zhang J; Liu M; Lin Y; Zhang R; Inhaled Tire-Wear Microplastic Particles Induced Pulmonary Fibrotic Injury via Epithelial Cytoskeleton Rearrangement. Environ. Int 2022, 164, 107257. 35486965 10.1016/j.envint.2022.107257

[111] Romero F; Shah D; Duong M; Penn RB; Fessler MB; Madenspacher J; Stafstrom W; Kavuru M; Lu B; Kallen CB; A Pneumocyte–Macrophage Paracrine Lipid Axis Drives the Lung toward Fibrosis. Am. J. Respir. Cell Mol. Biol 2015, 53, 74–86. 25409201 10.1165/rcmb.2014-0343OC PMC4566113

[112] Ogger PP; Byrne AJ Macrophage Metabolic Reprogramming during Chronic Lung Disease. Mucosal Immunol. 2021, 14, 282–295. 33184475 10.1038/s41385-020-00356-5 PMC7658438

[113] Laskin DL; Malaviya R; Laskin JD Role of Macrophages in Acute Lung Injury and Chronic Fibrosis Induced by Pulmonary Toxicants. Toxicol. Sci 2019, 168, 287–301. 30590802 10.1093/toxsci/kfy309 PMC6432864

[114] Hiraiwa K; van Eeden SF Contribution of Lung Macrophages to the Inflammatory Responses Induced by Exposure to Air Pollutants. Mediators Inflamm. 2013, 2013, 619523. 24058272 10.1155/2013/619523 PMC3766602

[115] Wong J; Magun BE; Wood LJ Lung Inflammation Caused by Inhaled Toxicants: A Review. Int. J. Chron. Obstruct. Pulmon. Dis 2016, 11, 1391–1401. 27382275 10.2147/COPD.S106009 PMC4922809

[116] Lin W-C; Fessler MB Regulatory Mechanisms of Neutrophil Migration from the Circulation to the Airspace. Cell. Mol. Life Sci 2021, 78, 4095–4124. 33544156 10.1007/s00018-021-03768-z PMC7863617

[117] Grommes J; Soehnlein O Contribution of Neutrophils to Acute Lung Injury. Mol. Med 2011, 17, 293–307. 21046059 10.2119/molmed.2010.00138 PMC3060975

[118] Keir HR; Chalmers JD Neutrophil Extracellular Traps in Chronic Lung Disease: Implications for Pathogenesis and Therapy. Eur. Respir. Rev 2022, 31, 210241. 35197267 10.1183/16000617.0241-2021 PMC9488971

[119] Jin YJ; Kim JE; Roh YJ; Song HJ; Seol A; Park J; Lim Y; Seo S; Hwang DY Characterisation of Changes in Global Genes Expression in the Lung of ICR Mice in Response to the Inflammation and Fibrosis Induced by Polystyrene Nanoplastics Inhalation. Toxicol. Res 2023, 39, 575–599. 10.1007/s43188-023-00188-y PMC10201517 37360972

[120] Ji Y; Chen L; Wang Y; Zhang J; Yu Y; Wang M; Wang X; Liu W; Yan B; Xiao L; Realistic Nanoplastics Induced Pulmonary Damage via the Crosstalk of Ferritinophagy and Mitochondrial Dysfunction. ACS Nano 2024, 18, 16790–16807. 38869479 10.1021/acsnano.4c02335

[121] Liu Y-Y; Liu J; Wu H; Zhang Q; Tang X-R; Li D; Li C-S; Liu Y; Cao A; Wang H Endocytosis, Distribution, and Exocytosis of Polystyrene Nanoparticles in Human Lung Cells. Nanomaterials 2023, 13, 84. 10.3390/nano13010084 PMC9824409 36615994

[122] Goodman KE; Hare JT; Khamis ZI; Hua T; Sang QXA Exposure of Human Lung Cells to Polystyrene Microplastics Significantly Retards Cell Proliferation and Triggers Morphological Changes. Chem. Res. Toxicol 2021, 34, 1069–1081. 33720697 10.1021/acs.chemrestox.0c00486

[123] Yang S; Zhang T; Ge Y; Cheng Y; Yin L; Pu Y; Chen Z; Liang G Ferritinophagy Mediated by Oxidative Stress-Driven Mitochondrial Damage Is Involved in the Polystyrene Nanoparticles-Induced Ferroptosis of Lung Injury. ACS Nano 2023, 17, 24988–25004. 38086097 10.1021/acsnano.3c07255

[124] Hwangbo S; Kim IY; Ko K; Park K; Hong J; Kang G; Wi J-S; Kim J; Lee TG Preparation of Fragmented Polyethylene Nanoplastics Using a Focused Ultrasonic System and Assessment of Their Cytotoxic Effects on Human Cells. Environ. Pollut 2024, 362, 125009. 39326828 10.1016/j.envpol.2024.125009

[125] Milillo C; Aruffo E; Di Carlo P; Patruno A; Gatta M; Bruno A; Dovizio M; Marinelli L; Dimmito MP; Di Giacomo V; Polystyrene Nanoplastics Mediate Oxidative Stress, Senescence, and Apoptosis in a Human Alveolar Epithelial Cell Line. Front. Public Health 2024, 12, 1385387. 38799687 10.3389/fpubh.2024.1385387 PMC11116779

[126] El Hayek E; Castillo E; In JG; Garcia M; Cerrato J; Brearley A; Gonzalez-Estrella J; Herbert G; Bleske B; Benavidez A; Photoaging of Polystyrene Microspheres Causes Oxidative Alterations to Surface Physicochemistry and Enhances Airway Epithelial Toxicity. Toxicol. Sci 2023, 193, 90–102. 36881996 10.1093/toxsci/kfad023 PMC10176241

[127] Luo H; Xiao T; Sun X; Song Y; Shi W; Lu K; Chen D; Sun C; Bian Q The Regulation of circRNA_kif26b on Alveolar Epithelial Cell Senescence via miR-346–3p Is Involved in Microplastics-Induced Lung Injuries. Sci. Total Environ 2023, 882, 163512. 37084911 10.1016/j.scitotenv.2023.163512

[128] Chilosi M; Carloni A; Rossi A; Poletti V Premature Lung Aging and Cellular Senescence in the Pathogenesis of Idiopathic Pulmonary Fibrosis and COPD/Emphysema. Transl. Res 2013, 162, 156–173. 23831269 10.1016/j.trsl.2013.06.004

[129] Kuwano K; Araya J; Hara H; Minagawa S; Takasaka N; Ito S; Kobayashi K; Nakayama K Cellular Senescence and Autophagy in the Pathogenesis of Chronic Obstructive Pulmonary Disease (COPD) and Idiopathic Pulmonary Fibrosis (IPF). Respir. Investig 2016, 54, 397–406. 10.1016/j.resinv.2016.03.010 27886850

[130] Woldhuis RR; de Vries M; Timens W; van den Berge M; Demaria M; Oliver BGG; Heijink IH; Brandsma C-A Link between Increased Cellular Senescence and Extracellular Matrix Changes in COPD. Am. J. Physiol. Lung Cell. Mol. Physiol 2020, 319, L48–L60. 32460521 10.1152/ajplung.00028.2020

[131] Barnes PJ; Baker J; Donnelly LE Cellular Senescence as a Mechanism and Target in Chronic Lung Diseases. Am. J. Respir. Crit. Care Med 2019, 200, 556–564. 30860857 10.1164/rccm.201810-1975TR

[132] Gutiérrez-García J; Egea R; Barguilla I; Nymark P; García-Rodríguez A; Guyot B; Maguer-Satta V; Marcos R; Rubio L; Hernández A Long-Term Exposure to Real-Life Polyethylene Terephthalate Nanoplastics Induces Carcinogenesis In Vitro. Environ. Sci. Technol 2025, 59, 10891–10904. 40452141 10.1021/acs.est.5c01628 PMC12164274

[133] Hanahan D Hallmarks of Cancer: New Dimensions. Cancer Discov. 2022, 12, 31–46. 35022204 10.1158/2159-8290.CD-21-1059

[134] Zhang J; Du J; Liu D; Zhuo J; Chu L; Li Y; Gao L; Xu M; Chen W; Huang W; Polystyrene Microplastics Induce Pulmonary Fibrosis by Promoting Alveolar Epithelial Cell Ferroptosis through cGAS/STING Signaling. Ecotoxicol. Environ. Saf 2024, 277, 116357. 38677073 10.1016/j.ecoenv.2024.116357

[135] Pikuda O; Xu EG; Berk D; Tufenkji N Toxicity Assessments of Micro- and Nanoplastics Can Be Confounded by Preservatives in Commercial Formulations. Environ. Sci. Technol. Lett 2019, 6, 21–25.

[136] Petersen EJ; Barrios AC; Henry TB; Johnson ME; Koelmans AA; Montoro Bustos AR; Matheson J; Roesslein M; Zhao J; Xing B Potential Artifacts and Control Experiments in Toxicity Tests of Nanoplastic and Microplastic Particles. Environ. Sci. Technol 2022, 56, 15192–15206. 36240263 10.1021/acs.est.2c04929 PMC10476161

[137] Yang L; Cao L; Zhang X; Chu B Targeting Ferroptosis as a Vulnerability in Pulmonary Diseases. Cell Death Dis. 2022, 13, 649. 35882850 10.1038/s41419-022-05070-7 PMC9315842

[138] Xu W; Deng H; Hu S; Zhang Y; Zheng L; Liu M; Chen Y; Wei J; Yang H; Lv X Role of Ferroptosis in Lung Diseases. J. Inflamm. Res 2021, 14, 2079–2090. 34045882 10.2147/JIR.S307081 PMC8144020

[139] Wu Y; Wang J; Zhao T; Sun M; Xu M; Che S; Pan Z; Wu C; Shen L Polystyrene Nanoplastics Lead to Ferroptosis in the Lungs. J. Adv. Res 2024, 56, 31–41. 36933884 10.1016/j.jare.2023.03.003 PMC10834790

[140] Wu Q; Liu C; Liu D; Wang Y; Qi H; Liu X; Zhang Y; Chen H; Zeng Y; Li J Polystyrene Nanoplastics-Induced Lung Apoptosis and Ferroptosis via ROS-Dependent Endoplasmic Reticulum Stress. Sci. Total Environ 2024, 912, 169260. 38086481 10.1016/j.scitotenv.2023.169260

[141] Yang S; Zhang T; Ge Y; Yin L; Pu Y; Liang G Inhalation Exposure to Polystyrene Nanoplastics Induces Chronic Obstructive Pulmonary Disease-like Lung Injury in Mice through Multi-Dimensional Assessment. Environ. Pollut 2024, 347, 123633. 38423272 10.1016/j.envpol.2024.123633

[142] Ghadban C; García-Unzueta M; Agüero J; Martín-Audera P; Lavín BA; Guerra AR; Berja A; Aranda N; Guzun A; Insua AI; Associations between Serum Levels of Ferroptosis-Related Molecules and Outcomes in Stable COPD: An Exploratory Prospective Observational Study. Intern. Emerg. Med 2025, 20, 1761–1773. 40542968 10.1007/s11739-025-04016-z PMC12476405

[143] Traversa A; Mari E; Pontecorvi P; Gerini G; Romano E; Megiorni F; Amedei A; Marchese C; Ranieri D; Ceccarelli S Polyethylene Micro/Nanoplastics Exposure Induces Epithelial–Mesenchymal Transition in Human Bronchial and Alveolar Epithelial Cells. Int. J. Mol. Sci 2024, 25, 10168. 39337653 10.3390/ijms251810168 PMC11432389

[144] Halimu G; Zhang Q; Liu L; Zhang Z; Wang X; Gu W; Zhang B; Dai Y; Zhang H; Zhang C; Toxic Effects of Nanoplastics with Different Sizes and Surface Charges on Epithelial-to-Mesenchymal Transition in A549 Cells and the Potential Toxicological Mechanism. J. Hazard. Mater 2022, 430, 128485. 35739668 10.1016/j.jhazmat.2022.128485

[145] Prudkin L; Liu DD; Ozburn NC; Sun M; Behrens C; Tang X; Brown KC; Bekele BN; Moran C; Wistuba II Epithelial-to-Mesenchymal Transition in the Development and Progression of Adenocarcinoma and Squamous Cell Carcinoma of the Lung. Mod. Pathol 2009, 22, 668–678. 19270647 10.1038/modpathol.2009.19 PMC2675657

[146] Milara J; Peiró T; Serrano A; Cortijo J Epithelial to Mesenchymal Transition Is Increased in Patients with COPD and Induced by Cigarette Smoke. Thorax 2013, 68, 410–420. 23299965 10.1136/thoraxjnl-2012-201761

[147] Li X; Zhang T; Lv W; Wang H; Chen H; Xu Q; Cai H; Dai J Intratracheal Administration of Polystyrene Microplastics Induces Pulmonary Fibrosis by Activating Oxidative Stress and Wnt/β-Catenin Signaling Pathway in Mice. Ecotoxicol. Environ. Saf 2022, 232, 113238. 35121255 10.1016/j.ecoenv.2022.113238

[148] Fan Z; Xiao T; Luo H; Chen D; Lu K; Shi W; Sun C; Bian Q A Study on the Roles of Long Non-Coding RNA and Circular RNA in the Pulmonary Injuries Induced by Polystyrene Microplastics. Environ. Int 2022, 163, 107223. 35390562 10.1016/j.envint.2022.107223

[149] Lim D; Jeong J; Song KS; Sung JH; Oh SM; Choi J Inhalation Toxicity of Polystyrene Micro(Nano)Plastics Using Modified OECD TG 412. Chemosphere 2021, 262, 128330. 33182093 10.1016/j.chemosphere.2020.128330

[150] Li L; Xu Y; Li S; Zhang X; Feng H; Dai Y; Zhao J; Yue T Molecular Modeling of Nanoplastic Transformations in Alveolar Fluid and Impacts on the Lung Surfactant Film. J. Hazard. Mater 2022, 427, 127872. 34862107 10.1016/j.jhazmat.2021.127872

[151] Cao Y; Zhao Q; Jiang F; Geng Y; Song H; Zhang L; Li C; Li J; Li Y; Hu X; Interactions between Inhalable Aged Microplastics and Lung Surfactant: Potential Pulmonary Health Risks. Environ. Res 2024, 245, 117803. 38043900 10.1016/j.envres.2023.117803

[152] Shi W; Cao Y; Chai X; Zhao Q; Geng Y; Liu D; Tian S Potential Health Risks of the Interaction of Microplastics and Lung Surfactant. J. Hazard. Mater 2022, 429, 128109. 35236033 10.1016/j.jhazmat.2021.128109

[153] Yang S; Cheng Y; Chen Z; Liu T; Yin L; Pu Y; Liang G In Vitro Evaluation of Nanoplastics Using Human Lung Epithelial Cells, Microarray Analysis and Co-Culture Model. Ecotoxicol. Environ. Saf 2021, 226, 112837. 34619472 10.1016/j.ecoenv.2021.112837

[154] Hu J; Wang C; Ma R; Qi S; Fu W; Zhong J; Cao C; Zhang X; Liu G; Gao Y Co-Exposure to Polyethylene Microplastics and House Dust Mites Aggravates Airway Epithelial Barrier Dysfunction and Airway Inflammation via CXCL1 Signaling Pathway in a Mouse Model. Int. Immunopharmacol 2025, 146, 113921. 39732106 10.1016/j.intimp.2024.113921

[155] Wu Q; Li R; You Y; Cheng W; Li Y; Feng Y; Fan Y; Wang Y Lung Microbiota Participated in Fibrous Microplastics (MPs) Aggravating OVA-Induced Asthma Disease in Mice. Food Chem. Toxicol 2024, 190, 114776. 38851522 10.1016/j.fct.2024.114776

[156] Xu M; Chen J; Gao L; Cai S; Dong H Microplastic Exposure Induces HSP90α Secretion and Aggravates Asthmatic Airway Remodeling via PI3K-Akt-mTOR Pathway. Ecotoxicol. Environ. Saf 2025, 291, 117828. 39923560 10.1016/j.ecoenv.2025.117828

[157] Lu K; Lai KP; Stoeger T; Ji S; Lin Z; Lin X; Chan TF; Fang JK-H; Lo M; Gao L; Detrimental Effects of Microplastic Exposure on Normal and Asthmatic Pulmonary Physiology. J. Hazard. Mater 2021, 416, 126069. 34492895 10.1016/j.jhazmat.2021.126069

[158] Wang C; Wu W; Pang Z; Liu J; Qiu J; Luan T; Deng J; Fang Z Polystyrene Microplastics Significantly Facilitate Influenza A Virus Infection of Host Cells. J. Hazard. Mater 2023, 446, 130617. 36623344 10.1016/j.jhazmat.2022.130617

[159] Liu Y; Møller P; Roursgaard M Aminated Polystyrene and DNA Strand Breaks in A549, Caco-2, THP-1 and U937 Human Cell Lines. Mutat. Res. Genet. Toxicol. Environ. Mutagen 2025, 903, 503865. 40185540 10.1016/j.mrgentox.2025.503865

